# Do you know what you’re looking for? Guiding templates are imprecise for both simple and real-world stimuli

**DOI:** 10.3758/s13414-026-03369-7

**Published:** 2026-11-11

**Authors:** Jessica N. Goetz, Mark B. Neider

**Affiliations:** Department of Psychology, University of Central Florida, 4111 Pictor Lane, Psychology Bldg 99, Ste. 320, Orlando, FL 32816-1390 USA

**Keywords:** Target templates, Visual search, Feature guidance, Eye movements, Selective attention

## Abstract

**Abstract:**

Recent research has found evidence that a highly veridical representation of the target is not used to guide attention. Rather, the template is broadly tuned to guide attention to a wider range of feature values than characterized by the target. In three experiments, we investigated whether a veridical representation of target features is used to guide attention when the target is a real-world object. In Experiment 1, we examined the imprecision of the guiding template when searching for real-world objects. We compared the coarseness of the guiding template across colored circles and real-world objects in Experiment 2. In Experiment 3, we evaluated if the accuracy of the encoded target item affected the coarseness of the guiding template. We found evidence across all three experiments that the guiding template does not represent the target veridically and that the coarseness of the template is affected by the initial precision of the target representation. The results support prior research suggesting that a coarser template may be inherent to efficient guidance regardless of stimulus type.

**Open Practices Statement:**

The data for all three experiments are available in the Open Science Framework (OSF) repository (https://doi.org/10.17605/OSF.IO/PBG4U). All three experiments were preregistered and can be found in the same repository.

## Introduction

How precise does the representation of your friend have to be when searching for them in a crowd? In order to guide our attention to search for an object, target information must be represented to inform the deployment of attentional processes. Recent work has found that an exact representation does not guide attention. Rather, a coarser representation does, but this has only been demonstrated when searching for colored circles (Yu et al., 2022). Here, we expand upon that previous work to investigate if a similarly coarse representation of the target information is used to guide attention for both more complex real-world objects and simple shapes.

Though many frameworks have been developed to understand and describe search processes (e.g., Oliva et al., 2003; Wolfe, 2021; Zelinsky, 2008), they generally share common concepts. Visual information about the environment is processed to create a spatial representation of the physical attributes. These attributes are basic visual features such as color, orientation, and size. The observer’s goal, specifically their target, is also represented in a conceptual template. Researchers have termed this a target, search, or guiding template with various nuanced definitions (e.g., Carlisle et al., 2011; Wolfe, 2021). For the purposes of this article, we use the term “guiding template” and “target template” as described by Wolfe (2021). A guiding template can be a representation of many features of many targets and is not resolved to a single specific target that is held in working memory. A target template is the representation of features for a specific target held in long-term memory. The guiding template informs and increases the sensory gain to target-consistent features, therefore increasing the priority signal to specific locations. Attention then selects the location with the highest attentional priority to determine if that was the target. If not, attention shifts to locations in descending priority. In summary, the mechanism used to search for an object uses a combination of information on the observer’s goals and the current visual environment. Though there are many factors that can influence search behavior, a critical focus of investigation is how and what visual features are used to guide attention.

Throughout the years, researchers have explored what types of information are stored in the guiding template. Wolfe and Horowitz (see 2004, 2017, for reviews) have compiled lists of features that are more or less likely to be represented in a template and used to guide search. Certain features such as color and size are considered “undoubted guiding features” due to the substantial evidence that attention is guided by such features. Generally, it has been found that certain features are selected to guide search over others. For example, color is frequently used even when other features are available to guide search (Alexander et al., 2019; Motter & Belky, 1998; Rutishauser & Koch, 2007). Recent work by Goetz and Neider (2025b) has expanded upon the dominance of color-based guidance. In their study, targets were either rectangles or real-world objects defined predominantly by two colors (e.g., red and green rectangle, red rose with a green stem). They, again, found evidence that the visual system prioritized guidance to color over shape when searching for real-world objects. Critically, the more informative or defining color was used to guide search over the other color. Their results indicate that the attentional system will prioritize the most informative feature to guide attention.

Some researchers have recently proposed the principle of “good-enough guidance,” which posits that while attention will use the most efficient feature, this feature is often imprecise or an approximation of the target feature (see Yu et al., 2023, for a review). More specifically, while the target template may represent target information more veridically, the guiding template’s representation is “good enough.”

A study conducted by Yu and colleagues (2022) provided critical evidence of good-enough guidance. Participants completed three different tasks: a memory task, a search decision task, and a guidance probe task. The target object was consistent across tasks and was defined as a single specific colored circle. In the memory task, participants had to indicate the closest color wedge on a color wheel to the target object’s color. In the search decision task, four colored circles, one target and three distractors, were presented and participants had to indicate the location of a notch on the target circle. Critically, one distractor termed the “critical distractor” could be any color within ± 100° rotation from the target color in steps of 10° (e.g., 10°, −20°, 90°). Like the search decision trials, the guidance probe trials began with a display of four colored circles, but after 120 ms, letters appeared in the circles and participants were asked to report all the letters they saw. Their results from the memory task indicated that the target template was indeed highly specific, with only approximately 2–3° of imprecision, but the guidance probe task indicated that the guiding template was coarser than the information used during the memory task. They reported that it was approximately 8–26° coarser, as attention was often directed to critical distractors that were rotated within this range. In other words, the information used to guide attention may be approximately 10–29° larger than the exact target feature value. However, the number of target-identification errors from the search decision task indicated that participants were more precise during identification than guidance. The authors concluded that the coarser guiding template optimizes the attentional system’s ability to shift to objects in the periphery, especially since there is a low cost to attending and rejecting non-target objects.

The goal of the current study was to extend the principle of good-enough guidance to more realistic search scenarios. Specifically, we examined if a similarly coarse guiding template is used to guide search for real-world objects. Researchers have previously posited that a coarser template is advantageous for shifting the attentional system toward peripheral locations (Yu et al., 2022, 2023). Additionally, a coarser template is effective when visual search occurs across different lighting conditions and scene contexts. Often, real-world objects are not defined by a single specific, consistent color. A pair of jeans, for example, can be defined by many colors of blue, due to imperfections in the fabric and different lighting conditions. Since Goetz and Neider (2025b) found evidence that the search process for real-world objects is likely similar to that for rectangles, it is likely that the mechanism described by most frameworks of visual search applies to both complex and simple stimuli. Yu and colleagues (2022) found that color information is represented more coarsely in the guiding template for circles, therefore we expected to find evidence that color information is also represented more coarsely in the guiding template for real-world objects.

## Experiment 1

In Experiment 1, we investigated whether the guiding template during search for real-world objects is coarser than a veridical representation of the target object. Our study largely replicated the search conditions of previous work (Yu et al., 2022): one critical distractor presented among several neutral distractors. Therefore, we expected attention to be tuned to colors approximately 10–29° in rotation from the veridical color and that it may be more beneficial to use an even coarser guiding template given the irregularities in color that often occur within the target object.

### Method

The experiment’s design and analyses were preregistered (https://doi.org/10.17605/OSF.IO/BCV6S). The experiment’s data can be found in the same repository.

#### Participants

Though our experiment is modeled closely on previous studies (Goetz & Neider, 2025a, b), the lowest effect size reported in those studies (η_p_^2^ = .83) with power of.95, α error probability of.025, one group, and eight measurements using the SPSS effect-size specification for a fully within-subjects repeated-measures ANOVA indicated only three participants were needed. Due to several design changes and to be more conservative, we chose to use a medium effect size (f = .25), power of.8, α error probability of.05, one group, and eight measurements using the SPSS effect size specification. The results of the power analysis indicated that 35 participants were required for adequate power. We recruited from the University of Central Florida Psychology Department subject pool until we had a final sample of 35 participants that met our inclusion criteria.

Participants were excluded if they performed at or below chance (≤ 50% accuracy) or had a mean response time (RT) ± 3 *SD* from the overall group mean RT. In total, we recruited 36 participants and one participant was removed for having a mean RT of above 3 *SD*s from the overall group mean RT. The final sample consisted of 17 females (18 males) with a mean age of 19.49 years. All participants had normal or corrected-to-normal visual acuity (20/30 on the Snellen chart for far vision, 20/32 on the Sloan ETDRS near vision) and color vision (12/13 plates on the Ishihara’s test for color blindness).

The research was approved by the Institutional Review Board at the University of Central Florida and complied with the American Psychological Association Code of Ethics. Informed consent was obtained from each participant, and each participant was granted partial course credit for the completion of the experiment.

#### Design and stimuli

The experiment was a 2 (target presence: absent, present) × 4 (critical distractor distance (CDD): 0°, 15°, 30°, 45°) fully within-subjects design. Each search display consisted of eight objects arranged in an invisible circle with a radius of 6.3° of visual angle. Objects were equally spaced from one another. For each search display, the circle of objects was randomly rotated such that the objects were not shown in the exact same eight locations.

**Fig. 1:**
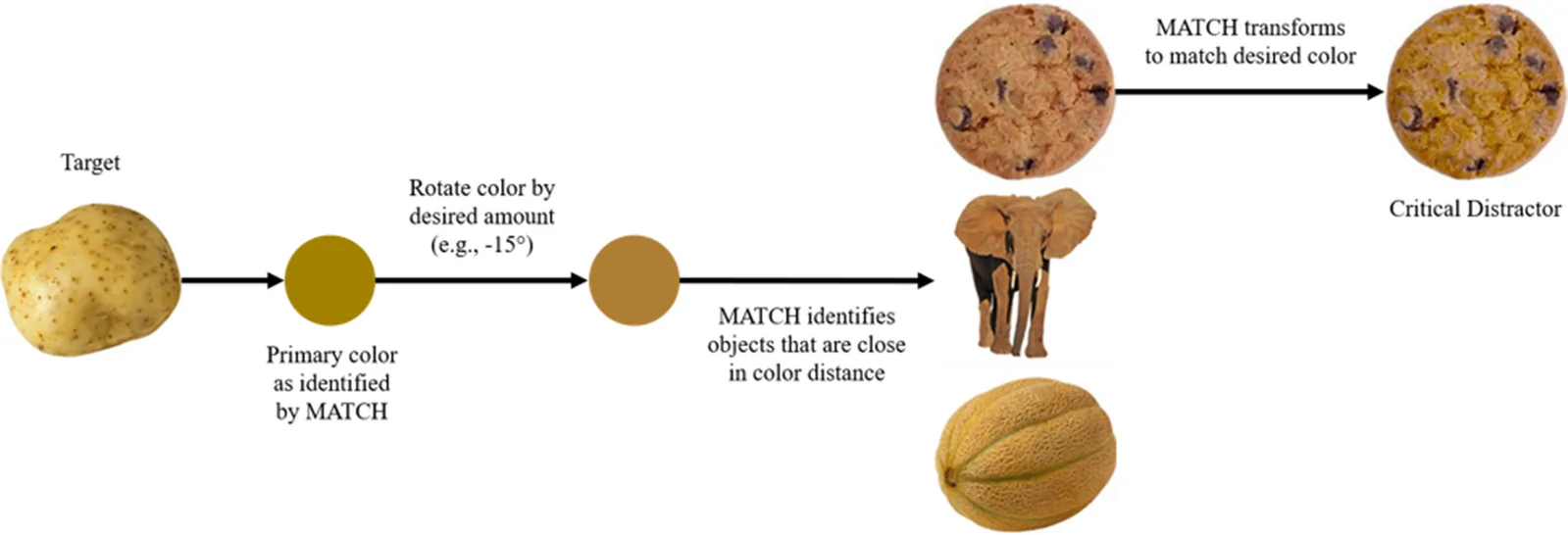
Graphical representation of using the MATCH (Matching and Transforming Closely Hued Objects) toolbox to create critical distractors

Stimuli were images of real-world objects procured from various stimulus databanks (Brady et al., 2008, 2013; Brodeur et al., 2014 (BOSS 2.0); Konkle et al., 2010). Since all the stimuli were images of real-world objects, the size could vary but all images were resized such that no image was larger than 2.70° × 2.70°. We used the MATCH (Matching and Transforming Closely Hued Objects) toolbox, which can objectively determine the color information within a real-world object and transform stimuli to match the color specified by the researcher (Goetz & Neider, 2025a). More specifically, the toolbox allows researchers to determine what color predominantly defines the object, which we will refer to as the object’s primary color. The toolbox can then take other stimuli and shift the colors within the stimulus to exactly match the object’s primary color. We used this process to generate critical color-matching distractors. The MATCH toolbox requires several inputs prior to the generation of color-matching stimuli. We designated that target and color-matching distractors had a 50% pixel coverage and the primary color was set as the filter color(s). In other words, the pixels of each stimulus were composed of at least 50% of the primary color. To generate distractors at specific rotations, we first determined the target’s primary color CIELUV values. We then applied the desired rotation of ± 15°, ± 30°, or ± 45°. The resulting color value was input into the MATCH toolbox to find a distractor with the nearest color. The distractor was then transformed to be the exact desired color value. CDDs were counterbalanced such that half were rotated positively while the remaining half were rotated negatively. An illustration of the process for generating critical color-matching distractor stimuli is shown in Fig. 1.

**Fig. 2:**
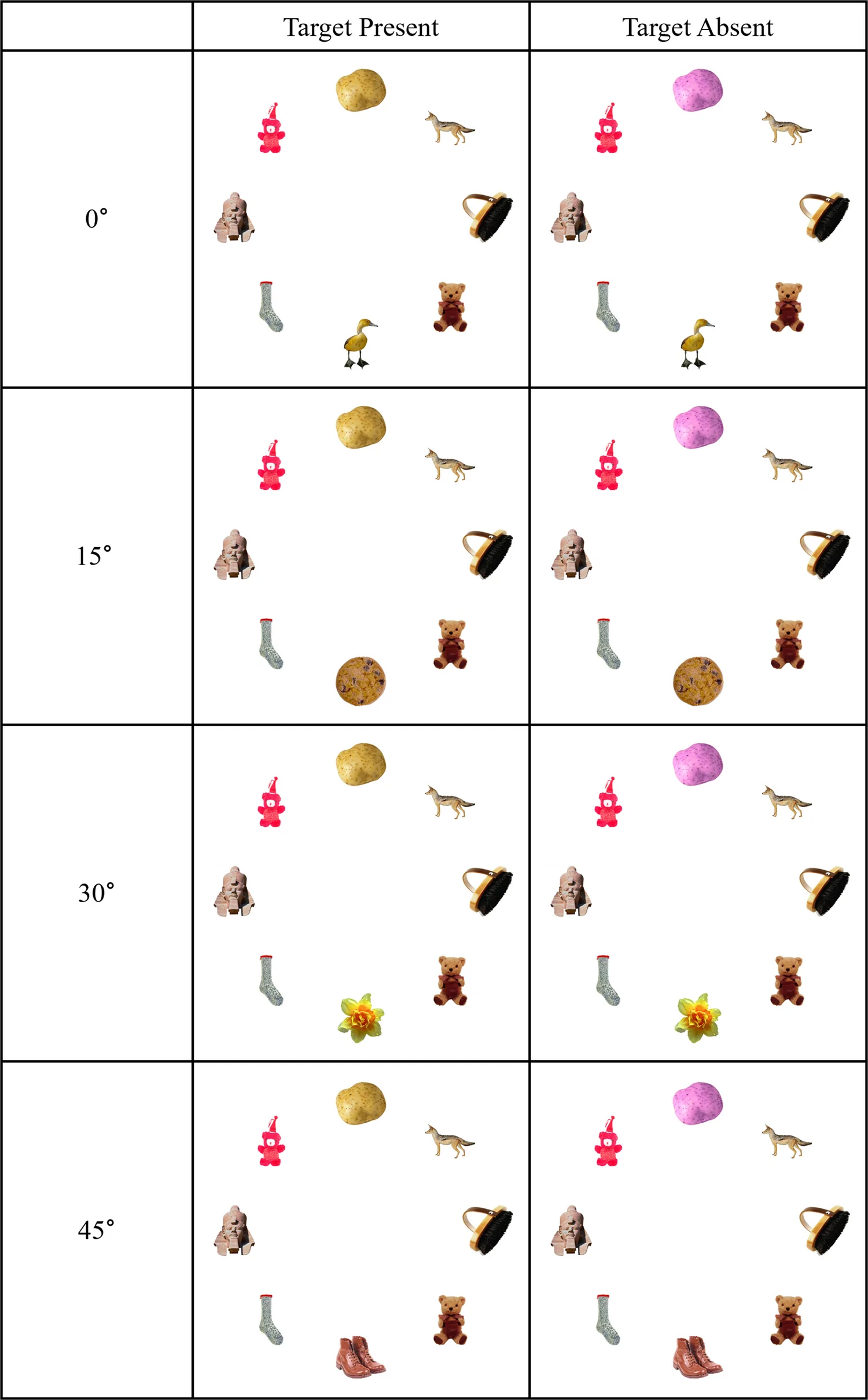
Example of each search array. In this example, the target was a potato, and the rotated target distractor was the potato rotated 261° in CIELUV color space and appeared on target-absent trials (presented in the top position for each search array). The critical distractor is presented in the bottom position for each search array. Additionally, the 15° array presents a critical distractor with a −15° rotation, the 30° array presents a critical distractor with a +30° rotation, and the 45° array presents a critical distractor with a −45° rotation. In the actual study, these objects could appear in any of the eight locations, the notional circle of eight objects was jitter-rotated so that they did not always appear in the exact location, and each array’s critical distractor rotation could be positive or negative

**Fig. 3:**
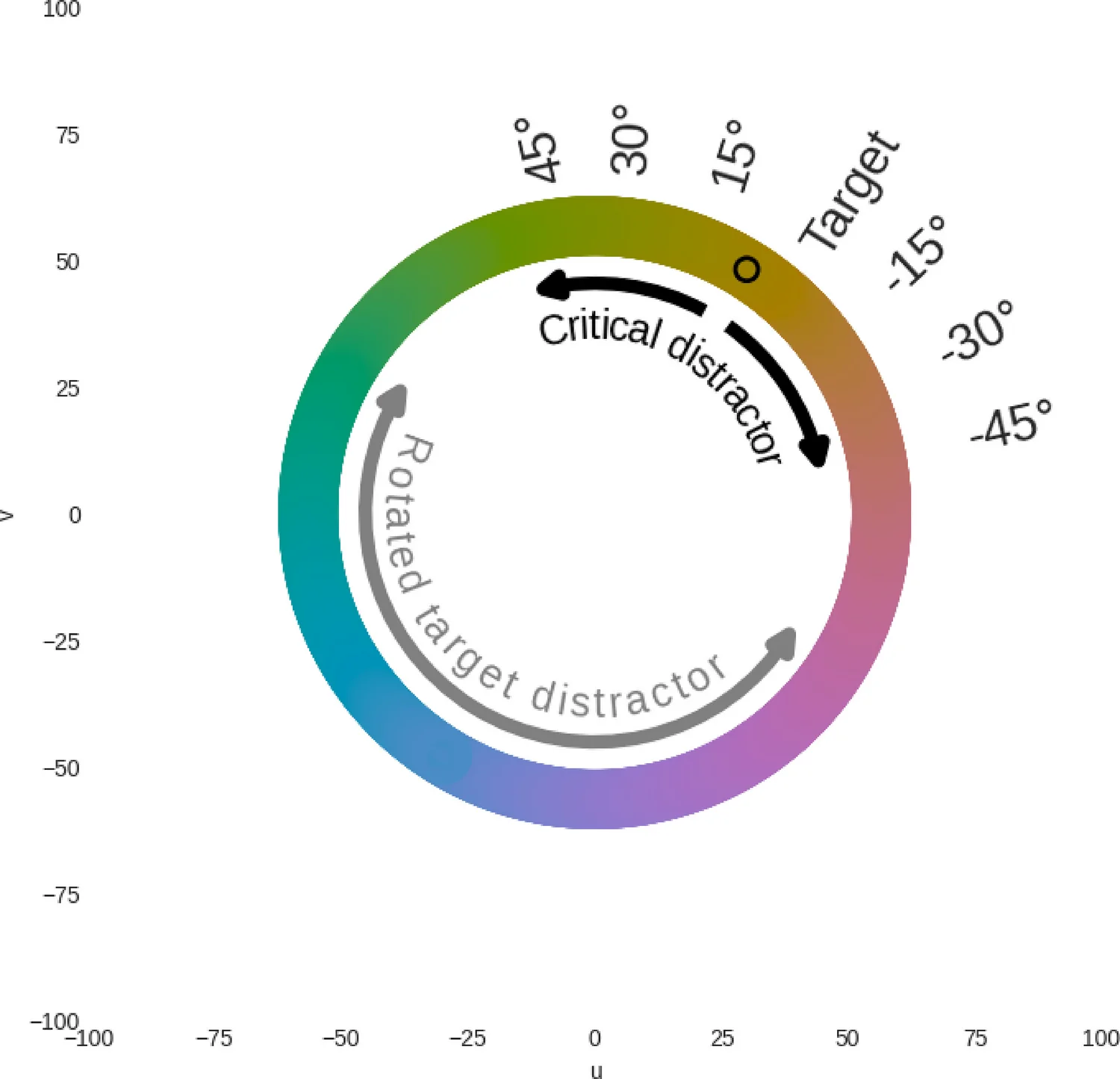
Color space for example target, critical distractor, and rotated target distractor. In this example, the target’s (u, v) was (30, 48), but each trial presented a different real-world target and therefore different (u, v) values defined each target

In addition to the critical color distractors, we also created a distractor that matched in shape, but not color, to appear in lieu of the target on target-absent trials. To generate these rotated target distractors, we took the target and applied a random rotation that could be any number between 90° and 270° in CIELUV color space. The selected rotation was applied to every pixel to generate a rotated target distractor. An example rotated target distractor can be found in Fig. 2 and a summary of the color space that a potential target, critical distractor, and rotated target distractor occupies can be found in Fig. 3.

Each target-present search array consisted of one target, one critical distractor, and six neutral distractors. Each target-absent search array consisted of one rotated target distractor, one critical distractor, and six neutral distractors. The different CDDs were evenly and randomly distributed such that there was a total of eight possible search arrays: target-present-0° critical distractor, target-present-15° critical distractor, target-present-30° critical distractor, target-present-45° critical distractor, target-absent-0° critical distractor, target-absent-15° critical distractor, target-absent-30° critical distractor, and target-absent-45° critical distractor. An illustration of each type of array is presented in Fig. 2.

#### Apparatus

Participants sat approximately 93.5 cm from a 24-in. Asus monitor with 1,920 × 1,080 resolution (53.5 cm × 30.0 cm). Their head movement was restricted by a chin and head rest. An EyeLink 1000 or EyeLink 1000 Plus eye-tracking system (SR Research Ltd) was used to record the participants’ right eye at 500 Hz. The experiment was programmed in Python and PsychoPy3 (Peirce et al., 2019). Participants made their responses via keyboard and mouse.

#### Procedure

Participants first provided verbal informed consent before they were prescreened for normal or corrected-to-normal vision. Participants had to score at least 20/30 on the Snellen chart for far vision, 20/32 on the Sloan ETDRS near vision, and at least 12/13 plates on the Ishihara’s test for color blindness. Prior to the experiment, participants completed a demographics survey. Participants were then provided with instructions on the task. They were asked to report on the presence of a target and to be as accurate and efficient in their responses as possible. Before the task began, participants were adjusted to sit comfortably in the chin and head rest. They then completed a 9-point calibration process using their right eye.

**Fig. 4:**
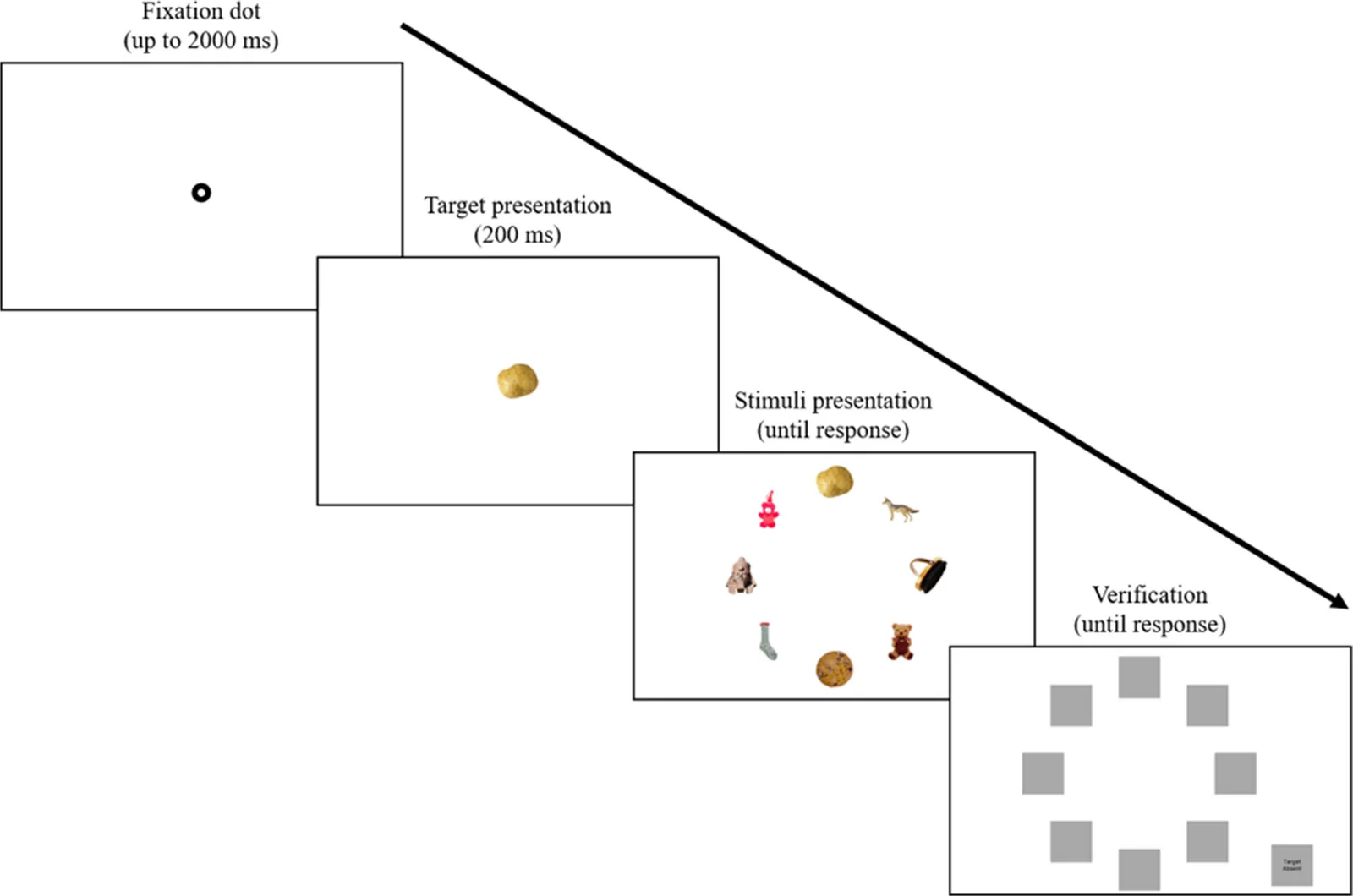
Example trial sequence

An example of each trial sequence is shown in Fig. 4. Each trial was preceded by a central fixation dot that subtended 0.10° of visual angle. Participants were required to continuously fixate the dot for 1,000 ms of a possible 2,000 ms or a manual drift check was triggered. If the manual drift check indicated an error above 2.00° of visual angle, the participant was recalibrated with a 9-point calibration. After the fixation dot, a central preview of the target for the upcoming trial appeared for 200 ms. The target differed for each trial. Following the target preview, a search display appeared and remained until the participant was ready to make a response. The participant pressed the space bar and the search display was replaced with gray boxes with an additional box in the bottom right corner of the screen that was labeled “Target absent.” Participants then verified the location (or absence) of the target by using the mouse to click on the appropriate box.

The experiment consisted of 16 practice trials where feedback was provided and 408 experimental trials where no feedback was provided. Half of the trials were target-present trials and the remaining half were target-absent trials. An equal number of trials for each CDD appeared. In other words, 51 of each trial type appeared (e.g., 51 target-present-0° critical distractor trials).

### Results and discussion

Data were prepared for analysis in R (Version 4.5.1) then analyzed through JASP (Version 0.18.1; JASP Team). All figures generated in R used the package ggplot2 (Wickham, 2016). Prior to all analyses, individual trials with any loss of eye movements (e.g., off-screen fixations) were removed. Across all participants, 50 trials (0.34% of all trials) were removed. For all the following ANOVA analyses, a Greenhouse-Geisser correction was employed when sphericity was violated. For all significant interactions, a simple main-effects analysis was conducted.

Our primary measure of attentional guidance was oculomotor behavior. Eye movements have been found to be highly coupled to shifts of attention (Henderson et al., 1989; Scialfa & Joffe, 1998; Williams et al., 1997). Since attention shifts first to a location with the highest priority (see Wolfe, 2021, for a review), a location that likely matches the features held in the guiding template, we used the proportion of initial saccades to objects of interest. We predicted that most initial saccades would be directed to the target on target-present trials. On target-absent trials, we predicted that most initial saccades would direct to the critical distractor, but as CDD increased, the proportion of initial saccades to the critical distractor would decrease. To provide supporting evidence that attention was guided to these objects, we conducted an additional exploratory analysis comparing each proportion of initial saccades to the chance of randomly saccading to each object.^1^ We conducted one-tailed *t*-tests for each proportion to chance (0.125). Additionally, we conducted a comparison of the proportion of initial saccades at CDD 45° between the critical distractor and neutral distractors to examine if the guiding template in our study was similar to the pattern of results reported in previous studies (Yu et al., 2022). If the guiding template is tuned to about 10–29° in rotation from the veridical color, we would expect the proportion to be similar between the critical distractor at CDD 45° and neutral distractors. In addition to oculomotor behavior, we used coarser measures of accuracy and RTs to further contextualize search performance. We expected performance to be worse on target-absent trials. We also expected that when the CDD was small (i.e., 0° or 15°), performance would be worse as a coarse template may guide attention to either the target or critical distractor.

#### Behavioral analysis

Participants were considered accurate on a trial if they localized the target object when it was a target-present trial or correctly selected the “Target absent” box when it was a target-absent trial. Accuracy was then determined by how many times they were correct divided by the total number of trials. RTs were measured from stimulus onset to when the participant hit “space,” indicating that they were ready to either localize the target or indicate that the trial was Target absent. The time spent localizing the target or selecting the “Target absent” box was not considered part of RTs.

#### Accuracy

We conducted a 2 (target presence) × 4 (CDD) repeated-measures ANOVA for accuracies. We present the mean accuracies in Table 1. As we expected, we found a main effect of target presence that was driven by higher accuracies on target-present than target-absent trials (*F*(1, 29) = 12.10, *p* = .002, η_p_^2^ = .29). There was also a main effect of CDD (*F*(3, 87) = 13.09, *p* <.001, η_p_^2^ = .31), so we conducted post hoc tests using a Bonferroni correction with a corrected alpha of.008. We found that accuracy was significantly higher when the CDD was 30° compared to 0°, 15°, and 45° (all *p*s < .001). There were no other significant pairwise comparisons (all *p*s ≥ .17).

**Table 1:** Means and standard deviations of accuracies in Experiment 1

	Target presence			
	Target present		Target absent	
Critical distractor distance	*M*	*SD*	*M*	*SD*
0°	98.10%	5.75%	95.09%	2.66%
15°	98.82%	3.94%	94.38%	3.77%
30°	98.37%	3.99%	98.36%	1.55%
45°	98.62%	2.83%	93.79%	3.64%

We also found a significant interaction between target presence and CDD (*F*(3, 87) = 17.54, *p* < .001, η_p_^2^ = .38). We found that there was a significant difference for accuracies on target-absent trials (*F*(3, 27) = 29.03, *p* < .001, η_p_^2^ = .76) but not target-present trials (*p* = .18, η_p_^2^ = .16). Specifically, accuracy was higher when the critical distractor was 30° compared to when it was 0°, 15°, and 45° (all *p*s < .001). All other comparisons were not significant (all *p*s ≥ .54). We expected performance to get better as the CDD increased. We found that accuracy was relatively high and stable across CDD.

#### Response times

**Fig. 5:**
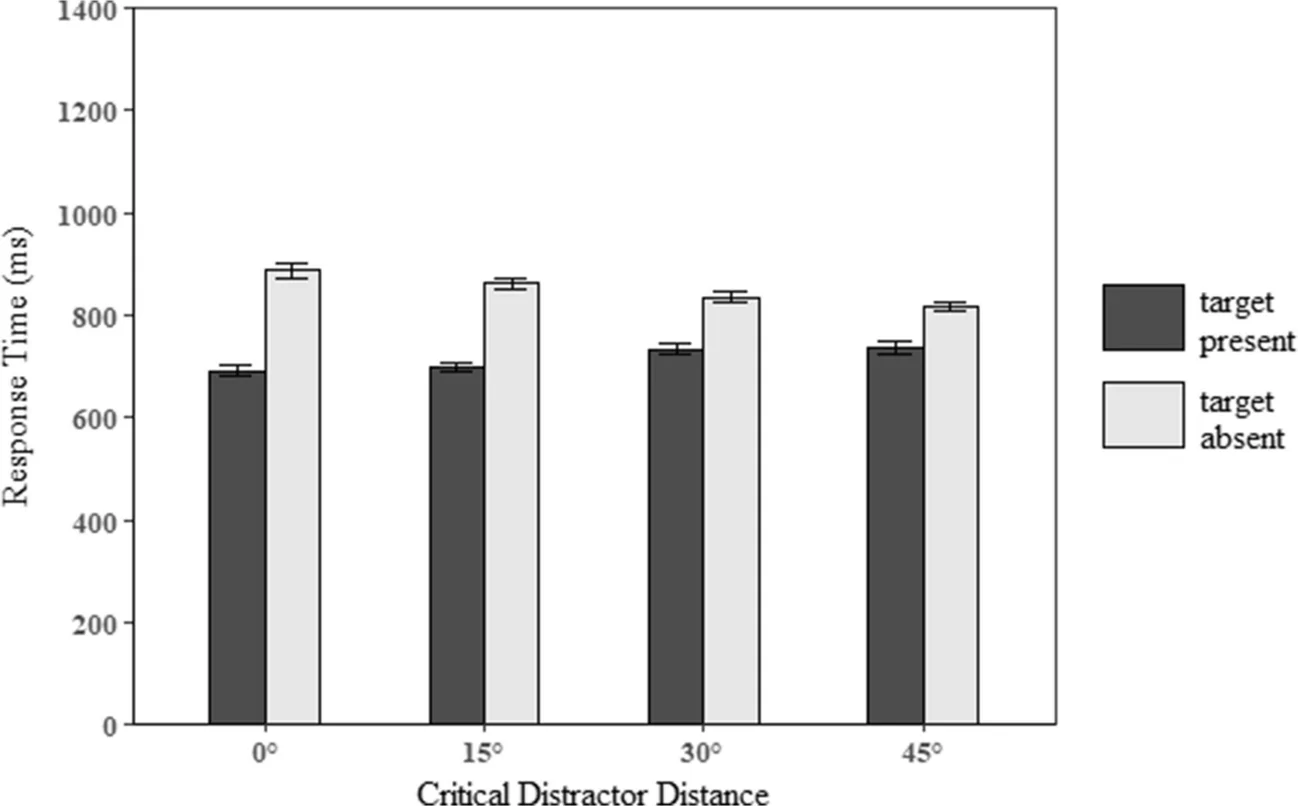
Response times for critical distractor distance by target presence in Experiment 1. Error bars represent two within-subjects standard errors above and below the mean using the Cousineau-Morey method (Morey, 2008)

A 2 × 4 repeated-measures ANOVA was conducted for RTs and the data are summarized in Fig. 5. There was a main effect of target presence such that participants were ~135 ms faster on target-present trials compared to target-absent trials (*F*(1, 29) = 72.67, *p* < .001, η_p_^2^ = .72). We found no main effect of CDD (*p* = .11, η_p_^2^ = .07). While RTs were expected to be faster on target-present trials, we initially expected that smaller CDDs would increase RTs as the guiding template would be directed to either the target or critical distractor.

While the non-significant main effect of CDD implied that the critical distractor had no capacity to distract, the significant interaction between target presence and CDD suggested a nuanced account (*F*(3, 87) = 19.87, *p* < .001, η_p_^2^ = .41). There was an unexpected significant difference on target-present trials (*F*(3, 27) = 5.14, *p* = .006, η_p_^2^ = .36). For target-present trials, pairwise comparisons indicated that participants were slower as CDD increased. Specifically, participants were significantly slower when the critical distractor was 30° compared to 0° and 15° (all *p*s ≤ .01) and when the critical distractor was 45° compared to 0° and 15° (all *p*s ≤ .04). We did find the predicted increase in RTs on target-absent trials as CDD increased (*F*(3, 27) = 15.38, *p* < .001, η_p_^2^ = .63), Participants were significantly faster when the CDD was 45° compared to 0, 15°, and 30° (all *p*s ≤ .04) and participants were significantly faster when the CDD was 30° compared to 0° (*p* = .004). All other comparisons between CDDs for target-present trials and target-absent trials were not significant (all *p*s ≥ .27).

#### Oculomotor analysis

To determine which object was attended to first, we used the initial saccade of each trial. Interest areas were defined as boxes that bound each object subtending 2.73° of visual angle. Initial saccade location was assigned to the object if the endpoint was in its bounding box. If the endpoint was not in a bounding box, we used the shortest Euclidian distance to a bounding box to determine the object attended. Since the sum of the proportion for each trial is 1.00, a main effect of CDD is not interpretable and will not be reported.

#### Target present

**Fig. 6:**
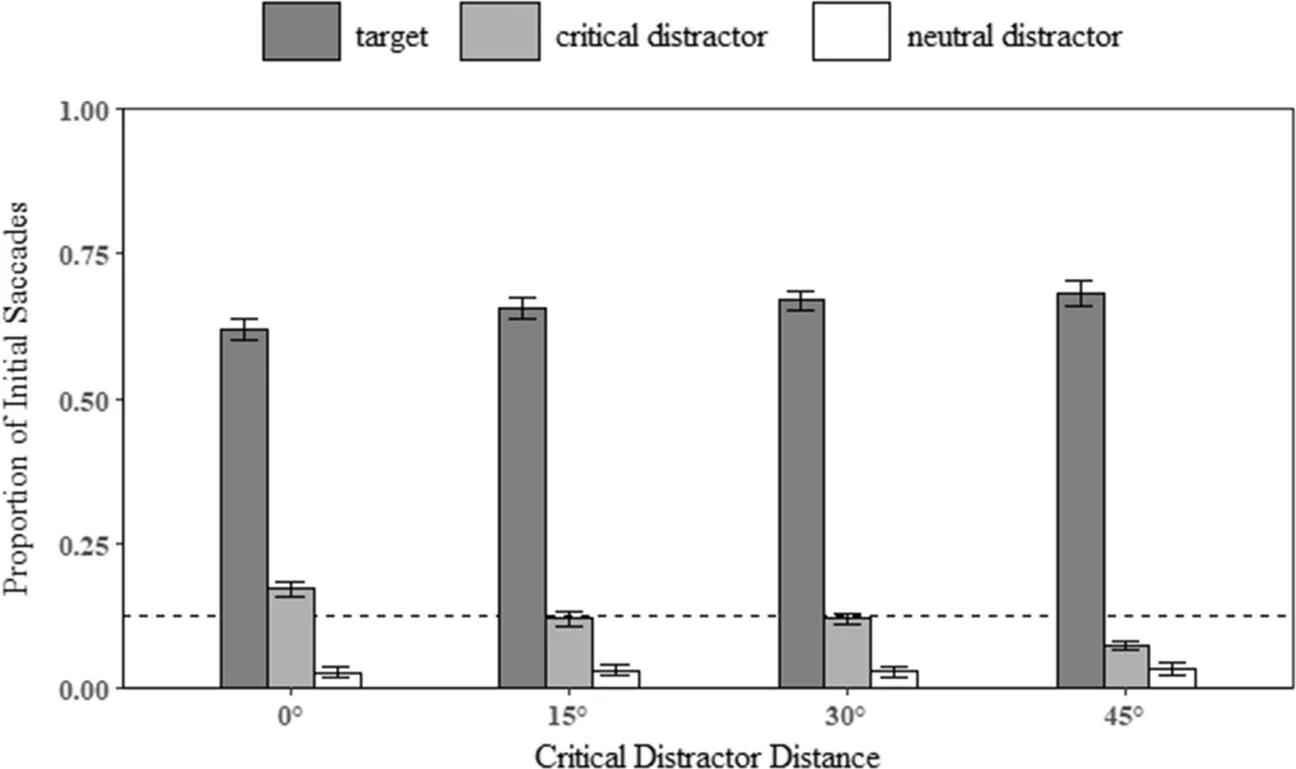
Proportion of initial saccades for object type by critical distractor distance on target-present trials in Experiment 1. The dotted line indicates the chance proportion if saccades were random (0.125). Error bars represent two within-subjects standard errors above and below the mean using the Cousineau-Morey method (Morey, 2008)

We conducted a 4 (CDD: 0°, 15°, 30°, 45°) × 3 (object type: target, critical distractor, neutral distractor) repeated-measures ANOVA for proportion of initial saccades to each object (see Fig. 6). We found a main effect of object type (*F*(1.04, 30.28) = 411.42, *p* < .001, η_p_^2^ = .93). To characterize the main effect, we used pairwise comparisons with a Bonferroni correction with a corrected alpha of.017. All pairwise comparisons were significant (all *p*s <.001). Most initial saccades were directed to the target followed by the critical distractor and then the neutral distractor, indicating that attention was highly efficient at locating the target.

We also found a significant interaction between CDD and object type (*F*(3.66, 106.24) = 13.70, *p* < .001, η_p_^2^ = .32). There were significant differences in the proportion of initial saccades for the target ((*F*(3, 27) = 5.28, *p* = .005, η_p_^2^ = .37) and critical distractor (*F*(3, 27) = 31.80, *p* < .001, η_p_^2^ = .78) but not the neutral distractor (*p* = .10, η_p_^2^ = .20). For initial saccades directed to the target, the proportion of initial saccades appeared to increase as CDD increased. Specifically, when the CDD was 0°, the proportion of initial saccades was smaller compared to when the CDD was 30° or 45° (all *p*s ≤ .03). Conversely, the proportion of initial saccades to the critical distractor appeared to decrease as CDD decreased. All pairwise comparisons were significant such that the proportion of initial saccades was largest when CDD was 0° followed by 15°, 30°, and then 45° (all *p*s ≤ .02). The results across the proportion of initial saccades directed to the target and critical distractor indicate that, while there was no significant difference in the gross measures of behavior, guidance was not always directed to the target at smaller CDDs. Instead, some proportion of initial saccades was directed to the critical color-matching distractor.

One-tailed *t*-tests for each proportion were compared to chance (0.125), with the results reported in Table 2. We found that the proportion of initial saccades directed to the target at all CDDs (0°, 15°, 30°, and 45°) and the critical distractor at CDD 0° were significantly above chance (all *p*s ≤ .001). All other proportions were not significant (all *p*s ≥ .56). The results provided supplemental support that attention was largely guided to the targets but occasionally a small proportion of saccades were guided to the critical distractor when the CDD was 0°.

**Table 2:** One-tailed *t*-test results comparing proportion of initial saccades on target present trials to chance in Experiment 1

Object type	Critical distractor distance	Test	Statistic	*df*	*p*-value	Effect size
Target	0°	Wilcoxon	464		<.001	>.99
	15°	Wilcoxon	464		<.001	>.99
	30°	Wilcoxon	464		<.001	>.99
	45°	Wilcoxon	464		<.001	>.99
Critical distractor	0°	Student	3.28	29	.001	0.60
	15°	Student	−0.31	29	.62	−0.06
	30°	Student	−0.14	29	.56	−0.03
	45°	Student	−9.73	29	>.99	−1.78
Neutral distractor	0°	Student	−33.03	29	>.99	−6.03
	15°	Student	−29.35	29	>.99	−5.36
	30°	Student	−33.32	29	>.99	−6.08
	45°	Student	−24.53	29	>.99	−4.48

The effect size reported for a Student’s *t*-test was Cohen’s *d.* The effect size reported for a Wilcoxon test was rank-biserial correlation (*r*)

#### Target absent

**Fig. 7:**
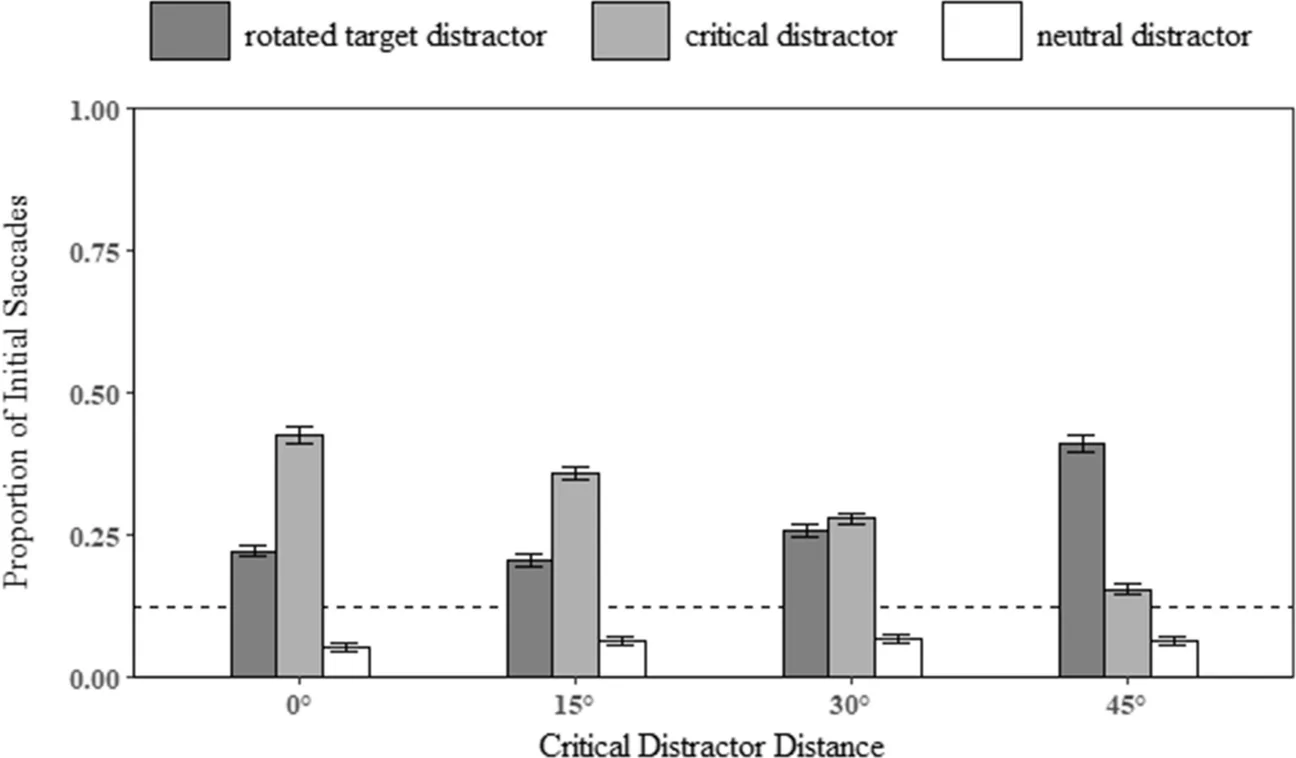
Proportion of initial saccades for object type by critical distractor distance on target absent trials in Experiment 1. The dotted line indicates the chance proportion if saccades were random (0.125). Error bars represent two within-subjects standard errors above and below the mean using the Cousineau-Morey method (Morey, 2008)

We present the data from the target-absent trials in Fig. 7. We conducted a 4 (CDD: 0°, 15°, 30°, 45°) × 3 (object type: rotated target distractor, critical distractor, neutral distractor) repeated-measures ANOVA for proportion of initial saccades to each object. There was a significant main effect of object type (*F*(2, 58) = 180.90, *p* < .001, η_p_^2^ = .86), so we conducted pairwise comparisons using a Bonferroni correction with a corrected alpha of.017. We found that there was a significantly larger proportion of initial saccades to the critical distractor compared to the neutral distractor (*p* < .001) and there was a significantly larger proportion of initial saccades to the rotated target distractor compared to the neutral distractor (*p* < .001). There was no significant difference in proportions between the critical distractor and rotated target distractor (*p* = .12). These results indicate that attention may be similarly guided to the distractor that shared the target’s color and the distractor that shared other feature dimensions to the target, such as shape.

We also found a significant interaction between CDD and object type (*F*(3.58, 103.80) = 107.46, *p* < .001, η_p_^2^ = .79). For initial saccades directed to the neutral distractor (*F*(3, 27) = 12.68, *p* < .001, η_p_^2^ = .59), there was a significantly smaller proportion of initial saccades when the CDD was 0° compared to 15°, 30°, and 45° (all *p*s < .001). For initial saccades directed to the critical distractor (*F*(3, 27) = 80.34, *p* < .001, η_p_^2^ = .90), we found all comparisons were significant, such that the proportion of initial saccades was largest when CDD was 0° followed by 15°, 30°, and then 45° (all *p*s ≤ .002). The linear decrease in proportion of initial saccades as CDD increases indicates that while the guiding template is coarse, the guiding template may not contain colors particularly far away from the target’s veridical color (e.g., 45°). Generally, we found that when the CDD was larger, there was a larger proportion of initial saccades directed to the rotated target distractor (*F*(3, 27) = 56.83, *p* < .001, η_p_^2^ = .86) Specifically, there was a significantly larger proportion of initial saccades when the CDD was 45° compared to when it was 0°, 15°, and 30° (all *p*s < .001). There was also a significantly larger proportion of initial saccades to the rotated target distractor when the CDD was 30° compared to 15° (*p* = .002). All other pairwise comparisons for initial saccades directed to the rotated target distractor were not significant (all *p*s ≥ .06). Together, the analyses of the proportion of initial saccades directed to the critical distractor and rotated target distractor indicate that, as the CDD gets further, observers may be using other features to guide attention. Instead of using color information, there may be a switch to using shape information at CDD 30° and especially at CDD 45°.

We conducted further analyses to examine if the proportions of initial saccades were above chance (0.125). We report the results of the one-tailed *t*-tests for each proportion in Table 3. It revealed that the proportion of initial saccades directed to the critical distractor at all CDDs (0°, 15°, 30°, and 45°) and the rotated target distractor at all CDDs (0°, 15°, 30°, and 45°) were above chance (all *p*s ≤ .01). All other proportions were not significant (all *p*s = 1.00). The results provided additional support that attention was guided to objects that share some feature dimension with the target.

**Table 3:** One-tailed *t*-test results comparing proportion of initial saccades on target absent trials to chance in Experiment 1

Object type	Critical distractor distance	Test	Statistic	*df*	*p*-value	Effect size
Rotated target distractor	0°	Student	7.56	29	<.001	1.38
	15°	Student	5.23	29	<.001	0.95
	30°	Student	8.39	29	<.001	1.53
	45°	Student	13.51	29	<.001	2.47
Critical distractor	0°	Wilcoxon	464		<.001	>.99
	15°	Wilcoxon	463		<.001	.99
	30°	Wilcoxon	457		<.001	.97
	45°	Student	2.65	29	0.01	0.48
Neutral distractor	0°	Student	−22.99	29	>.99	−4.20
	15°	Student	−14.49	29	>.99	−2.65
	30°	Wilcoxon	0		>.99	<-.99
	45°	Student	−16.62	29	>.99	−3.03

The effect size reported for a Student’s *t*-test was Cohen’s *d.* The effect size reported for a Wilcoxon test was rank-biserial correlation (*r*)

#### 45° critical distractor versus neutral distractor

A *t*-test was conducted to compare the proportion of initial saccades to the critical distractor when the CDD was 45° to the neutral distractor for target-absent trials. We chose to only conduct the analysis on target-absent trials since most initial saccades were directed to the target on target-present trials. We found that the proportion of initial saccades was larger for the critical distractor compared to the neutral distractor (*t*(29) = 9.45, *p* <.001, *d* = 0.33). The results indicate that the critical distractor at CDD 45° is not comparable to a neutral distractor. Neutral distractors were objects that were at least 15 units of Euclidean distance in L*, u*, v* color space from the target’s color. Combined with the evidence that the proportion at CDD 45° for the critical distractor was above chance, the results indicate that search was guided by an even coarser template for color than the initially expected range of 10–29° when searching for real-world objects.

## Experiment 2

The results of Experiment 1 indicated that search was guided to critical distractors that were rotated as far as 45° from the target’s primary color. A coarser template than initially predicted may be related to the demands that often occur in the real world. Often, visual search occurs when there are changes in lighting conditions and the contextual surrounding. Additionally, real-world objects are rarely defined by a singular color value (e.g., a light brown potato presented in Fig. 1 encompasses a range of browns). To test the possibility that even coarser templates are used during search for real-world objects, we compared guidance for real-world objects and colored circles as previously used by Yu and colleagues (2022). We predicted that for the behavioral measures, we would find similar results to Experiment 1, where participants were faster and more accurate on target-present trials. If guidance was more broadly tuned for real-world objects compared to colored circles, we would expect to find a larger proportion of initial saccades to objects further in color space from the target’s primary color compared to when the observer searched for colored circles.

### Method

The study’s design and analysis plan were preregistered at https://doi.org/10.17605/OSF.IO/Y5EF7. The procedures were similar to Experiment 1 with the exceptions outlined below.

#### Participants

Using the effect size from significant interaction on target-absent trials in Experiment 1 (η_p_^2^ = .79) with power of.95, α error probability of.025, two groups, and six measurements using the SPSS effect size specification for a mixed repeated-measures ANOVA indicated that we would only need six participants for adequate power. As there were several critical design changes, a more conservative power analysis was conducted. We used a general medium effect size (f = .25), power of.8, α error probability of.05, two groups, and six measurements which indicated that we would need a total of 46 participants or 23 per between-subject condition. We recruited until we had 46 participants who met all our inclusion criteria. Those who participated in Experiment 1 were ineligible and our exclusion criteria were the same as Experiment 1: at or below chance performance (≤ 50% accuracy) and RTs ± 3 *SD* from the overall group mean RT. In total, we recruited 47 participants, as one participant had a mean RT above 3 *SD*s from the overall group mean RT. The final sample of 46 participants consisted of 28 females (18 males) with a mean of age 18.76 years.

#### Design and stimuli

With the inclusion of a between-subject factor of either real-world objects or colored circles, we now employed a mixed design. In addition, we had to remove the color distances of 0° because there would always be two colored circles with the exact color on target-present trials. Thus, the final design was a 2 (stimulus type: real-world objects, colored circles; between-subject) × 2 (target presence: present, absent; within-subject) × 3 (CDD: 15°, 30°, 45°; within-subject).

**Fig. 8:**
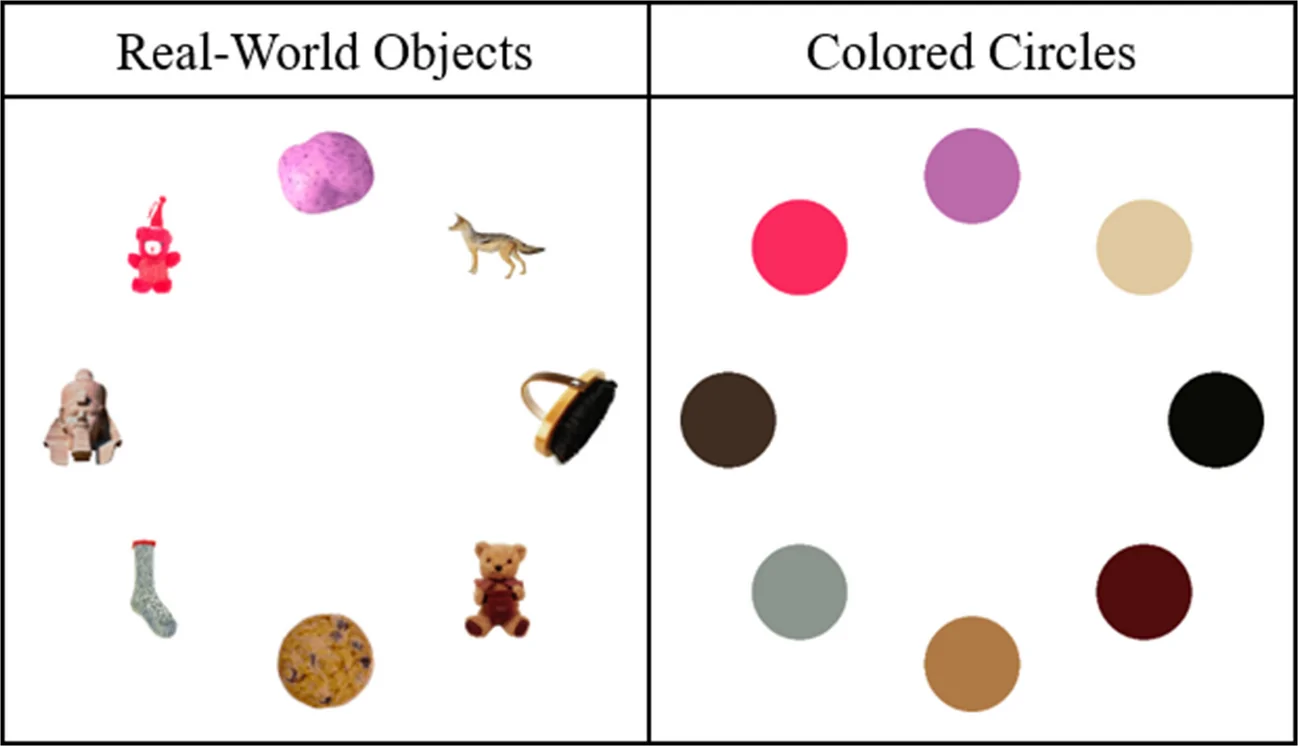
Example trials for real-world objects versus colored circles condition*.* In this example, for both stimulus type conditions, a target-absent trial with CDD of 15° (bottom object) is shown. The top object is characterized as the rotated target distractor to conduct statistical analyses across the two conditions

The same exact stimuli from Experiment 1 were used for the real-world objects condition. To control similarity in color, for the colored circles condition, we determined and used the primary color of every stimulus on a given trial presented in Experiment 1 by using the MATCH toolbox (Goetz & Neider, 2025a). An example of equivalent trials is shown in Fig. 8. In the real-world object condition, the rotated target distractor matched the target’s shape but not color. In the colored circles condition, since all objects matched in shape, the rotated target distractor would be indistinguishable from a neutral distractor. However, to compare performance across the between-subject conditions, we will still designate that circle as the rotated target distractor. For example, in Fig. 8, the purple potato was the rotated target distractor and an equivalent purple circle was shown in the colored circles condition.

#### Procedure

Participants completed the same task as in Experiment 1 in either the real-world objects or colored circles condition. However, there were now 12 practice trials where feedback was provided and 306 experimental trials where no feedback was provided in order to keep equivalent trials per trial type across the two experiments. Therefore, like Experiment 1, there were 51 of each trial type (e.g., 51 target-present-15° critical distractor trials).

### Results and discussion

Data were prepared as outlined in Experiment 1. We removed 13 trials (0.09% of all trials) for loss of eye-movement data. In addition, we conducted a planned comparison to investigate whether the template was coarser for real-world objects compared to colored circles. Specifically, we examined the proportion of initial saccades between stimulus type and the critical distractor and neutral distractor at CDD 45°.

#### Behavioral analysis

##### Accuracy

We conducted a 2 (stimulus type) × 2 (target presence) × 3 (CDD) mixed ANOVA for accuracy and the summary means are presented in Table 4. We found a significant main effect of stimulus type where the difference in accuracy indicated that the colored circles condition was significantly more difficult than the real-world objects condition (*F*(1, 44) = 391.98, *p* < .001, η_p_^2^ = .90). These findings are unsurprising as observers can use more than one feature to guide and determine the identity of the target in real-world objects. As expected, there was also a main effect of target presence such that participants were significantly more accurate on target-present trials compared to target-absent trials (*F*(1, 44) = 64.61, *p* <.001, η_p_^2^ = .59). There was also a significant main effect of CDD (*F*(1.69, 74.49) = 207.56, *p* < .001, η_p_^2^ = .83), so we conducted post hoc tests using a Bonferroni correction with a corrected alpha of.017. We found that accuracy was significantly lower when the CDD was 15° compared to when it was 30° and 45° (all *p*s < .001) and were no significant differences in accuracy between 30° and 45° (*p* = .06). As expected, when the CDD was smaller participants may have mistaken the critical distractor as the target.

**Table 4:** Means and standard deviations of accuracies for Experiment 2

		Target presence			
		Target present		Target absent	
Stimulus type	Critical distractor distance	*M*	*SD*	*M*	*SD*
Real-world objects	15°	99.40%	0.92%	94.71%	3.52%
	30°	98.98%	1.00%	98.03%	2.37%
	45°	99.31%	1.13%	92.84%	4.45%
Colored circles	15°	69.12%	9.23%	35.83%	14.42%
	30°	83.78%	6.97%	73.57%	11.82%
	45°	86.18%	8.07%	71.39%	10.34%

We found several significant interactions: stimulus type and target presence (*F*(1, 44) = 27.79, *p* < .001, η_p_^2^ = .39), stimulus type and CDD (*F*(1.69, 74.49) = 200.52, *p* < .001, η_p_^2^ = .82), target presence and CDD (*F*(1.58, 69.58) = 120.53, *p* <.001, η_p_^2^ = .73), and a three-way interaction (*F*(1.58, 69.58) = 85.95, *p* < .001, η_p_^2^ = .66). Since we were interested in the differences between the stimulus types, to characterize the three-way interaction, we conducted 2 (stimulus type) × 3 (CDD) ANOVAs for target-present and target-absent trials separately.

The interaction between stimulus type and target presence indicated that there were no significant differences in accuracy for real-world objects (*p* = .06, η_p_^2^ = .08). However, there was a significant effect for colored circles (*F*(1, 44) = 88.57, *p* < .001, η_p_^2^ = .67). Participants were more accurate on target-present trials than target-absent trials providing evidence that the participants likely had a difficult time discriminating the identity of the target between the actual target and the critical distractor.

For stimulus type and CDD, we found significant differences for both real-world objects (*F*(2, 43) = 4.47, *p* = .02, η_p_^2^ = .17) and colored circles (*F*(2, 43) = 278.32, *p* < .001, η_p_^2^ = .93). When searching for real-world objects, participants were significantly less accurate when the CDD was 45° compared to when it was 30° (*p* = .01). We found this result to be surprising. We would have expected accuracy to be lower when CDD is smaller. For colored circles, we found the expected pattern where participants were significantly less accurate when the CDD was 15° compared to when it was 30° and 45° (all *p*s < .001). Other pairwise comparison between CDDs for real-world objects and colored circles did not differ (all *p*s ≥ .65).

For target presence and CDD, we expected accuracy to be lower when CDD was smaller, indicating that participants may have misidentified the critical distractor that was similar in color to the target as the target. We found a significant difference in accuracies for both target-present trials (*F*(2, 43) = 40.88, *p* < .001, η_p_^2^ = .66) and target-absent trials (*F*(2, 43) = 220.05, *p* <.001, η_p_^2^ = .91). On target-present trials, participants were significantly less accurate when the CDD was 15° compared to when it was 30° or 45° (all *p*s <.001). There were no differences in accuracies between CDDs of 30° and 45° (*p* = .10). Likewise, on target-absent trials, participants were most accurate when the CDD was 45° followed by 15° and then 30° (all *p*s < .001).

For the three-way interaction, the separate 2 (stimulus type) × 3 (CDD) ANOVAs for target-present and target-absent trials revealed a significant interaction on target-present trials (*F*(1.65, 72.51) = 61.49, *p* < .001, η_p_^2^ = .58) and target-absent trials (*F*(1.68, 73.85) = 269.87, *p* < .001, η_p_^2^ = .86). On target-present trials, we found that participants were significantly more accurate when searching for real-world objects compared to colored circles when the CDD was 15° (*F*(1, 44) = 245.20, *p* < .001, η_p_^2^ = .85), 30° (*F*(1, 44) = 107.17, *p* < .001, η_p_^2^ = .71), and 45° (*F*(1, 44) = 59.76, *p* < .001, η_p_^2^ = .58). On target-absent trials, we found that participants were also significantly more accurate when searching for real-world objects compared to colored circles when the CDD was 15° (*F*(1, 44) = 361.75, *p* < .001, η_p_^2^ = .89), 30° (*F*(1, 44) = 94.66, *p* < .001, η_p_^2^ = .68), and 45° (*F*(1, 44) = 83.48, *p* < .001, η_p_^2^ = .66). In other words, the three-way interaction demonstrated that across target presence and CDD, while participants were consistently more accurate when searching for real-world objects compared to colored circles, the scale of accuracy differences just differed. Specifically, there was a greater decrement in accuracy between the two stimulus conditions on target-absent trials.

##### Response times

**Fig. 9:**
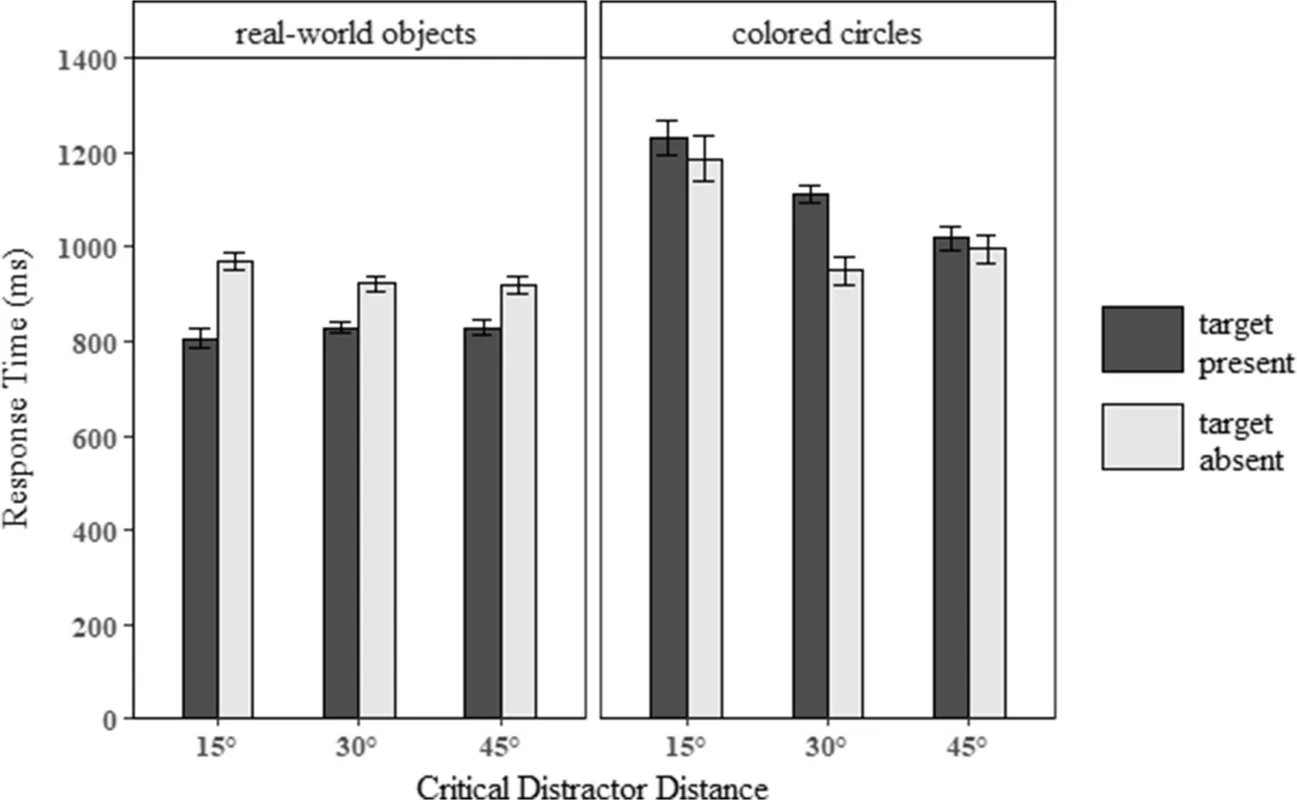
Response times for critical distractor distance by target presence for each stimulus type for Experiment 2. Error bars represent two within-subjects standard errors above and below the mean using the Cousineau-Morey method (Morey, 2008)

The means RTs are presented in Fig. 9. A 2 × 2 × 3 mixed ANOVA was conducted. We found a significant main effect of stimulus type such that participants were ~203 ms faster when participants searched for real-world objects compared to colored circles (*F*(1, 44) = 7.42, *p* = .009, η_p_^2^ = .14) providing further evidence that searching for real-world objects was easier compared to colored circles. There was no significant main effect of target presence (*p* = .34, η_p_^2^ = .02) but there was a significant main effect of CDD (*F*(1. 35, 59.21) = 20.41, *p* < .001, η_p_^2^ = .32). Using post hoc tests with a Bonferroni correction with a corrected alpha of.017 we found that participants were slower when the CDD was small. RTs may have been increased if the coarse guiding template directed attention to the critical distractor. We found that participants were significantly slower when the CDD was 15° compared to when it was 30° and 45° (all *p*s < .001). There were no RT differences between CDDs of 30° and 45° (*p* = .21).

We found several significant interactions: stimulus type and target presence (*F*(1, 44) = 21.04, *p* < .001, η_p_^2^ = .32), stimulus type and CDD (*F*(1.35, 59.21) = 15.52, *p* < .001, η_p_^2^ = .26), target presence and CDD (*F*(1.72, 75.63) = 6.87, *p* = .003, η_p_^2^ = .14), and a three-way interaction (*F*(1.72, 75.63) = 3.88, *p* = .03, η_p_^2^ = .08). For the three-way interaction, we separately conducted −2 (stimulus type) × 3 (CDD) ANOVAs for target-present and target-absent trials.

The interaction between stimulus type and target presence indicated participants were ~116 ms faster on target-present trials compared to target-absent trials when searching for real-world objects (*F*(1, 44) = 15.43, *p* < .001, η_p_^2^ = .26). Conversely, participants were ~76 ms slower on target-present trials compared to target-absent trials when searching for colored circles (*F*(1, 44) = 6.55, *p* = .01, η_p_^2^ = .13). While we expected RTs to typically be faster on target-present trials, participants likely had a difficult time discriminating between the critical distractor and target on target-present trials when searching for colored circles.

For stimulus type and CDD we found a significant difference for colored circles (*F*(2, 43) = 22.27, *p* <.001, η_p_^2^ = .51). Participants were significantly slower when the CDD was 15° compared to when it was 30° and 45° (all *p*s < .001), providing further evidence that participants had a difficult time discriminating between the critical distractor and target when searching for colored circles. There were no differences in RTs between CDDs of 30° and 45° (*p* = .34). We also did not find a difference in RTs across CDDs for real-world objects (*p* = .89, η_p_^2^ = .01). Although we did not find large behavioral differences across CDDs in Experiment 1, there were differences in oculomotor behavior across CDDs. Therefore, we expected to find differences across CDDs in the eye movements but not RTs.

For target presence and CDD, there was a significant effect for both target-present trials (*F*(2, 43) = 16.10, *p* < .001, η_p_^2^ = .43) and target-absent trials (*F*(2, 43) = 10.69, *p* < .001, η_p_^2^ = .33). Specifically, on target-present trials, participants were significantly faster when the CDD was 45° compared to when it was 15° and 30° (all *p* ≤ .002). On target-absent trials, participants were significantly slower when the CDD was 15° compared to when it was 30° and 45° (all *p*s < .001). Other pairwise comparison between CDDs for target-present and target-absent trials did not differ (all *p*s ≥ .08). Regardless of whether the target was present, participants were slower when CDDs were smaller, providing evidence that attention was less efficient with the presence of a critical distractor that was similar in color to the target object. We speculate that participants were guided towards objects close to the target’s veridical color, which slowed performance.

Separate 2 (stimulus type) × 3 (CDD) ANOVAs for target-present and target-absent trials were conducted for the three-way interaction. We found a significant interaction on target-present trials (*F*(1.59, 69.82) = 21.82, *p* < .001, η_p_^2^ = .33) and target-absent trials (*F*(1.41, 61.94) = 6.92, *p* = .005, η_p_^2^ = .14). When searching for real-world objects compared to colored circles on target-present trials, participants were ~425 ms faster when the CDD was 15° (*F*(1, 44) = 18.10, *p* < .001, η_p_^2^ = .29), ~281 ms faster when the CDD was 30° (*F*(1, 44) = 14.63, *p* < .001, η_p_^2^ = .25), and ~190 ms faster when the CDD was 45° (*F*(1, 44) = 6.12, *p* = .02, η_p_^2^ = .12). On target-absent trials, we found that participants were ~216 ms faster when searching for real-world objects compared to colored circles on target-absent trials when the CDD was 15° (*F*(1, 44) = 4.57, *p* = .04, η_p_^2^ = .09). There were no significant RT differences between stimulus types and when the CDD was 30° (*p* = .25, η_p_^2^ = .03) or 45° (*p* = .68, η_p_^2^ = .004). In summary, the three-way interaction indicated that participants were significantly faster at searching for the target when the stimulus was real-world objects, but there was less of a benefit on target-absent trials. Overall, searching for real-world objects would be easier as there are more features to use to guide attention compared to searching for colored circles.

##### Oculomotor analysis

Like Experiment 1, the sum of the proportion for each trial is 1.00, therefore a main effect of CDD is not interpretable. This also applies to each between-subject condition of stimulus type. Therefore, main effects of CDD and stimulus type or their interaction will not be reported.

##### Target present

**Fig. 10:**
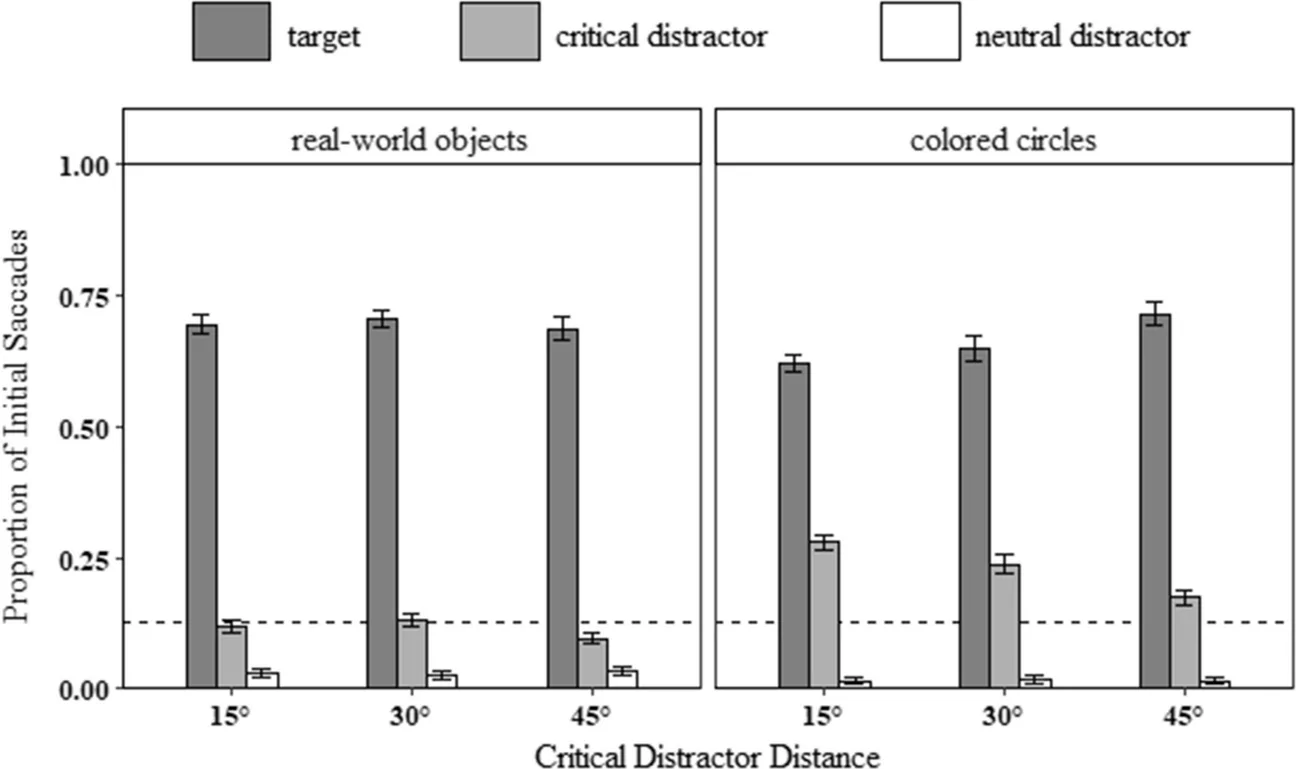
Proportion of initial saccades for object type by critical distractor distance on target present trials for each stimulus type for Experiment 2. The dotted line indicates the chance proportion if saccades were random (0.125). Error bars represent two within-subjects standard errors above and below the mean using the Cousineau-Morey method (Morey, 2008)

We conducted a 2 (stimulus type: real-world objects, colored circles) × 3 (CDD: 15°, 30°, 45°) x 3 (object type: target, critical distractor, neutral distractor) mixed ANOVA for proportion of initial saccades to each object. The mean proportions of initial saccades are presented in Fig. 10. We found a significant main effect of object type (*F*(1.13, 49.64) = 1127.30, *p* < .001, η_p_^2^ = .96), so we conducted post hoc tests with a Bonferroni correction using a corrected alpha of.017. We found that the proportion of initial saccades was largest for the target followed by the critical distractor and then neutral distractor (all *ps* < .001). Once again, we found that search was highly efficient at locating the target but was occasionally misguided to the critical distractor.

There were several significant interactions: stimulus type and object type (*F*(1.13, 49.64) = 15.81, *p* < .001, η_p_^2^ = .26), CDD and object type (*F*(2.17, 95.49) = 12.86, *p* < .001, η_p_^2^ = .23), and a three-way interaction (*F*(2.17, 95.49) = 9.81, *p* < .001, η_p_^2^ = .18). Critically, to explore how guidance was affected across stimulus type and CDD, for the three-way interaction, we conducted three separate 2 (stimulus type) × 3 (CDD) ANOVAs, one for each object type.

The analysis for stimulus type and object type revealed that while there was no significant difference in the proportion of initial saccades to the target object (*p* =.22, η_p_^2^ =.03), there was a significant difference for critical distractors (*F*(1, 44) = 85.53, *p* < .001, η_p_^2^ = .66) and neutral distractors (*F*(1, 44) = 13.75, *p* < .001, η_p_^2^ = .24). Specifically, there was a larger proportion of initial saccades to the critical distractor when participants were searching for colored circles compared to real-world objects but there was a larger proportion of initial saccades to the neutral distractor when searching for real-world objects compared to colored circles. Together, the results indicate that attention was directed to the target-similar distractors more often in the colored circles condition. Rather than a coarser guiding template for colored circles, it is likely that observers could use more than just the target’s color to effectively guide attention when searching for real-world objects.

For CDD and object type, we found there was a significant difference in the proportion of initial saccades for the target (*F*(2, 43) = 5.64, *p* = .007, η_p_^2^ = .21) and the critical distractor (*F*(2, 43) = 34.96, *p* < .001, η_p_^2^ = .62) but not the neutral distractor (*p* = .32, η_p_^2^ = .05). We expected search to be more efficient at locating the target when the CDD was large and found evidence to support that hypothesis. There was a significantly larger proportion of initial saccades to the target when the CDD was 45° compared to when it was 15° (*p* = .005). Likewise, fewer initial saccades were directed to the critical distractor when the CDD was 45° compared to when it was 15° and 30° (all *p*s < .001). All other pairwise comparisons between CDDs for the target and critical distractor did not differ (all *p*s ≥ .14).

For the three-way interaction, we conducted a 2 (stimulus type) × 3 (CDD) ANOVA for each object type. We found a significant interaction for when the initial saccade was directed to the target (*F*(2, 88) = 9.64, *p* < .001, η_p_^2^ = .18), critical distractor (*F*(1.73, 75.93) = 9.15, *p* = .001, η_p_^2^ = .17), and neutral distractor (*F*(2, 88) = 6.55, *p* = .002, η_p_^2^ = .13). There was a significantly larger proportion of initial saccades to the target when the stimulus type was real-world objects compared to colored circles at CDD 15° (*F*(1, 44) = 7.05, *p* =.01, η_p_^2^ =.14). There were no significant differences in the proportion of initial saccades to the target between stimulus types at CDDs 30° (*p* = .08, η_p_^2^ = .07) or 45° (*p* = .40, η_p_^2^ = .02). We found that when the initial saccades were directed to the critical distractor, the proportion was smaller when searching for real-world objects compared to colored circles at CDD 15° (*F*(1, 44) = 77.18, *p* < .001, η_p_^2^ = .64), 30° (*F*(1, 44) = 39.58, *p* < .001, η_p_^2^ = .47), and 45° (*F*(1, 44) = 27.01, *p* < .001, η_p_^2^ =.38). Conversely, when the initial saccades were directed to the neutral distractor, the proportion was larger when searching for real-world objects compared to colored circles at CDD 15° (*F*(1, 44) = 16.76, *p* < .001, η_p_^2^ = .28), 30° (*F*(1, 44) = 4.43, *p* = .04, η_p_^2^ = .09), and 45° (*F*(1, 44) = 16.47, *p* < .001, η_p_^2^ = .27). In summary, when searching for real-world objects, observers were more effective at locating the target in the first saccade at CDD 15°, but when searching for colored circles, observers frequently directed their first saccade to the critical distractor even for colored circles that were 45° away from the target’s color. Observers likely could use many different feature dimensions to locate the target in the real-world objects condition, increasing their efficiency. On the other hand, when searching for a colored circle, color was the only efficient guiding feature.

When we compared each proportion of initial saccades to chance (0.125), one-tailed *t*-tests (see Table 5) revealed that the proportion of initial saccades directed to the target at all CDDs (15°, 30°, and 45°) in the real-world objects condition, the target at all CDDs (15°, 30°, and 45°) in the colored circles condition, and the critical distractor at all CDDs (15°, 30°, and 45°) in the colored circles condition (all *p*s < .001). All other proportions were not significant (all *p*s ≥ .64). The results provide some additional context for the oculomotor behavior. In the real-world objects condition, there was little guidance to the critical distractor and search was highly efficient at locating the target. However, in the colored circles condition, we provide additional evidence that search was indeed often guided to the critical distractor providing evidence that the guiding template was coarse.

**Table 5:** One-tailed *t*-test results comparing proportion of initial saccades on target present trials to chance in Experiment 2

Stimulus type	Object type	Critical distractor distance	Test	Statistic	*df*	*p*	Effect size
Real-world objects	Target	15°	Student	25.84	22	<.001	5.39
		30°	Student	28.84	22	<.001	6.01
		45°	Student	22.90	22	<.001	4.78
	Critical distractor	15°	Student	−0.65	22	.74	−0.14
		30°	Wilcoxon	127		.64	-.08
		45°	Student	−3.63	22	>.99	−0.76
	Neutral distractor	15°	Student	−31.51	22	>.99	−6.57
		30°	Student	−30.81	22	>.99	−6.42
		45°	Student	−23.59	22	>.99	−4.92
Colored circles	Target	15°	Student	28.55	22	<.001	5.95
		30°	Wilcoxon	276		<.001	>.99
		45°	Student	25.64	22	<.001	5.35
	Critical distractor	15°	Student	10.14	22	<.001	2.11
		30°	Wilcoxon	276		<.001	>.99
		45°	Student	3.86	22	<.001	0.80
	Neutral distractor	15°	Student	−52.52	22	>.99	−10.95
		30°	Student	−39.76	22	>.99	−8.29
		45°	Wilcoxon	0		>.99	<-.99

The effect size reported for a Student’s *t*-test was Cohen’s *d.* The effect size reported for a Wilcoxon test was rank-biserial correlation (*r*)

#### Target absent

**Fig. 11:**
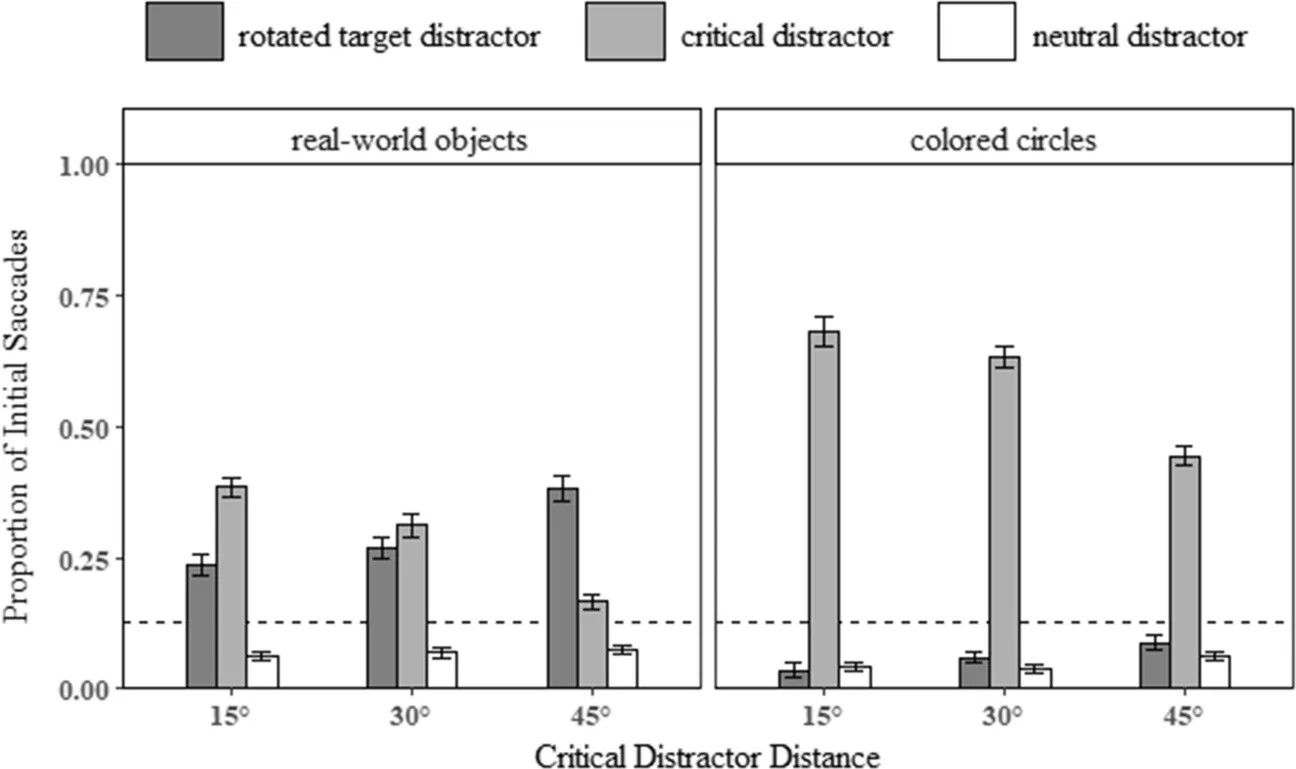
Proportion of initial saccades for object type by critical distractor distance on target absent trials for each stimulus type for Experiment 2. The dotted line indicates the chance proportion if saccades were random (0.125). Error bars represent two within-subjects standard errors above and below the mean using the Cousineau-Morey method (Morey, 2008)

We conducted a 2 (stimulus type: real-world objects, colored circles) × 3 (CDD: 15°, 30°, 45°) × 3 (object type: rotated target distractor, critical distractor, neutral distractor) mixed ANOVA for proportion of initial saccades to each object (see Fig. 11). There was a significant main effect of object type (*F*(1.44, 63.40) = 328.11, *p* < .001, η_p_^2^ = .88). Post hoc tests with a Bonferroni correction with a corrected alpha of.017 were conducted to investigate the main effect of object type. We found that most initial saccades were directed to the critical distractor followed by the rotated target distractor and then neutral distractor (all *p*s < .001). The results indicated that the search was most often guided to the color matching distractor.

We found several significant interactions: stimulus type and object type (*F*(1.44, 63.40) = 157.10, *p* < .001, η_p_^2^ = .78), CDD and object type (*F*(2.97, 130.67) = 91.15, *p* < .001, η_p_^2^ = .67), and a three-way interaction (*F*(2.97, 130.67) = 2.83, *p* = .04, η_p_^2^ = .06). Like target-present trials, we were interested in the effects of guidance across stimulus type and CDD. Therefore, we conducted separate 2 (stimulus type) × 3 (CDD) ANOVAs for each object type to further characterize the three-way interaction.

Further analysis between stimulus type and object type revealed a significant difference in the proportion of initial saccades that were directed to the critical distractor (*F*(1, 44) = 168.66, *p* <.001, η_p_^2^ =.79), rotated target distractor (*F*(1, 44) = 21.74, *p* < .001, η_p_^2^ = .33), and neutral distractors (*F*(1, 44) = 104.57, *p* < .001, η_p_^2^ = .70). Overall, the results suggest that color was the predominant feature used to guide attention when searching for colored circles, but other features, such as shape, were used to guide attention when searching for real-world objects. Specifically, there was a smaller proportion of initial saccades to the critical distractor when participants were searching for real-world objects compared to colored circles but there was a larger proportion of initial saccades to the rotated target distractor and neutral distractor when searching for real-world objects compared to colored circles.

For CDD and object type, we found that there was a significant difference in the proportion of initial saccades for the critical distractor (*F*(2, 43) = 124.26, *p* < .001, η_p_^2^ =.85), rotated target distractor (*F*(2, 43) = 31.67, *p* < .001, η_p_^2^ = .60), and neutral distractor (*F*(2, 43) = 20.87, *p* < .001, η_p_^2^ = .49). For the proportion of initial saccades that were directed to the neutral distractor, we found a significantly larger proportion was directed to the neutral distractor when the CDD was 45° compared to 15° and 30° (all *p*s <.001). There were no differences in the proportion of initial saccades to the neutral distractor at CDDs of 15° and 30° (*p* = 1.00). Meanwhile, the pattern of initial saccades directed to the critical distractor and rotated distractor indicated that as CDD increased, color did not predominantly guide attention. As CDD was smaller, more initial saccades were directed to the critical distractor but as CDD got larger, more initial saccades were directed to the rotated target distractor. Specifically, initial saccades were directed to the critical distractor most when the CDD was 15° followed by 30° and then 45° (all *p*s ≤ .007) but initial saccades were directed to the rotated target distractor most when the CDD was 15° followed by 30° and then 45° (all *p*s ≤ .03). However, this analysis collapses across stimulus conditions and no other features than color could guide attention in the colored circles condition. Further analysis of the three-way interaction is necessary.

For each object type, a 2 (stimulus type) × 3 (CDD) ANOVA was conducted for the three-way interaction. We did not find a significant interaction for initial saccades directed to the critical distractor (*p* = .43, η_p_^2^ = .02). However, we found a significant interaction for when the initial saccade was directed to the rotated target distractor (*F*(2, 88) = 9.81, *p* < .001, η_p_^2^ = .18) and neutral distractor (*F*(2, 88) = 4.97, *p* = .01, η_p_^2^ = .10). There was a significantly larger proportion of initial saccades to the rotated target distractor when the stimulus type was real-world objects compared to colored circles at CDDs 15° (*F*(1, 44) = 64.24, *p* < .001, η_p_^2^ = .59), 30° (*F*(1, 44) = 70.05, *p* < .001, η_p_^2^ = .61), and 45° (*F*(1, 44) = 96.88, *p* < .001, η_p_^2^ = .69). Likewise, when the initial saccades were directed to the neutral distractor, the proportion was larger when searching for real-world objects compared to colored circles at CDD 15° (*F*(1, 44) = 9.33, *p* = .004, η_p_^2^ = .18), 30° (*F*(1, 44) = 40.40, *p* < .001, η_p_^2^ = .48), and 45° (*F*(1, 44) = 4.82, *p* = .03, η_p_^2^ = .10). To summarize the analysis of the three-way interaction, we found that in the absence of the target, most initial saccades were directed to the critical distractor across stimulus types and CDDs. Critically, this provides evidence that coarse guiding templates were used when searching for both real-world objects and colored circles. However, in the real-world objects condition, participants appeared to use features other than color to aid search as many initial saccades were directed to the shape-matching object.

The results of the one-tailed *t*-tests comparing each proportion of initial saccades to chance (0.125) are presented in Table 6. We found that the proportion of initial saccades directed to the critical distractor at all CDDs (15°, 30°, and 45°) in the real-world objects condition, the critical distractor at all CDDs (15°, 30°, and 45°) in the colored circles condition, and the rotated target distractor at all CDDs (15°, 30°, and 45°) in the real-world objects condition were greater than chance (all *p*s < .001). All other proportions were not significant (all *p*s ≥ .996). The results provide additional evidence that search was guided to both the critical distractor and rotated target distractor when searching for real-world objects but only the critical distractor when searching for colored circles. Again, the results indicate that other feature dimensions than just color guided search for real-world objects.

**Table 6:** One-tailed *t*-test results comparing proportion of initial saccades on target absent trials to chance in Experiment 2

Stimulus type	Object type	Critical distractor distance	Test	Statistic	*Df*	*p-*value	Effect size
Real-world objects	Rotated target distractor	15°	Wilcoxon	241		<.001	.91
		30°	Student	6.36	22	<.001	1.33
		45°	Student	9.53	22	<.001	1.99
	Critical distractor	15°	Student	15.03	22	<.001	3.13
		30°	Student	8.26	22	<.001	1.72
		45°	Student	3.66	22	<.001	0.76
	Neutral distractor	15°	Student	−17.83	22	>.99	−3.72
		30°	Student	−13.86	22	>.99	−2.89
		45°	Student	−12.55	22	>.99	−2.62
Colored circles	Rotated target distractor	15°	Wilcoxon	17		>.99	-.88
		30°	Wilcoxon	11		>.99	-.92
		45°	Student	−2.88	22	>.99	−0.60
	Critical distractor	15°	Student	18.94	22	<.001	3.95
		30°	Student	22.40	22	<.001	4.67
		45°	Student	15.07	22	<.001	3.14
	Neutral distractor	15°	Student	−17.19	22	>.99	−3.58
		30°	Student	−35.08	22	>.99	−7.32
		45°	Student	−16.10	22	>.99	−3.36

The effect size reported for a Student’s *t*-test was Cohen’s *d.* The effect size reported for a Wilcoxon test was rank-biserial correlation (*r*)

#### 45° critical distractor versus neutral distractor

To investigate if a coarser template was employed, we conducted a 2 (stimulus type: real-world objects, colored circles) × 2 (object type: critical distractor, neutral distractor) mixed ANOVA. The analysis was constrained to target-absent trials only as most initial saccades were directed to the target on target-present trials. We focused on the interaction since the main effect is captured by the ANOVAs reported above. The interaction was significant (*F*(1, 44) = 121.54, *p* < .001, η_p_^2^ = .73). We found that there was a significantly larger proportion of initial saccades to the critical distractor compared to the neutral distractor for both stimulus types: real-world objects (*F*(1, 44) = 420.85, *p* < .001, η_p_^2^ = .91) and colored circles (*F*(1, 44) = 24.24, *p* < .001, η_p_^2^ = .36). Our results indicate that a coarse template was used to guide attention when searching for real-world objects and colored circles. We initially proposed that a coarser template was used for real-world objects compared to colored circles since search for real-world objects oftentimes occurs under more complex contexts. There may be differences in lighting conditions and real-world objects are rarely defined by a single specific, consistent color. Real-world objects are defined by many other features, and attention does not only use color as a guiding feature. In this study, participants may be strategically utilizing shape (or other features) to guide attention. However, the results suggest that an even coarser template was used to guide attention in the colored circles condition compared to previous research (Yu et al., 2022). In comparison to previous research, our study switched the target every trial which may have led to the difference in template coarseness.

## Experiment 3

Across Experiments 1 and 2, we found evidence that attention was often misguided to distractors that were similar in color to the target object. Our results provided additional support to the proposal that guiding templates consist of a broader set of features than the veridical representation of the target (Yu et al., 2022). Regardless of the stimulus we used, oculomotor behavior in our studies indicated that the guiding templates include colors that expand far beyond the originally proposed ~10–29° in color space. Unlike previous studies, the target changes every trial. Many studies have found evidence that a less accurate representation, and potentially coarser template is formed when targets switch trial-to-trial (e.g., Bravo & Farid, 2012; Goldstein & Beck, 2018). In these studies, they argued that the information moves from working memory to long-term memory. To that point, recent literature has demonstrated that objects that were more accurately represented in memory guided attention better (Williams et al., 2022). Therefore, the results across Experiments 1 and 2 could be due to a poor representation of the target object in long-term memory resulting in a coarse representation that is used as a guiding template. To resolve this, we used a single target and compared guidance across real-world objects and colored circles. Our goal in Experiment 3 was to investigate if coarser templates are used for real-world objects, even when the object is well-represented in memory.

### Method

The study’s design and analysis plan were preregistered at https://doi.org/10.17605/OSF.IO/45WB3. The procedure was similar to Experiment 2 with the exceptions outlined below.

#### Participants

We used the same sample size rational as Experiment 2 and recruited until we had 46 participants (23 per condition) who met all our inclusion criteria. Our exclusion criteria were the same as Experiments 1 and 2 (≤ 50% accuracy and RTs ± 3 *SD* from the overall group mean RT) and could not be participants who participated in Experiments 1 and 2. We recruited 47 participants as one participant had a mean RT above 3 *SD*s from the overall group mean RT. The final sample of 46 participants had a mean age of 19.46 and consisted of 25 females, 20 males, and one participant who preferred not to respond.

#### Design and stimuli

**Fig. 12:**
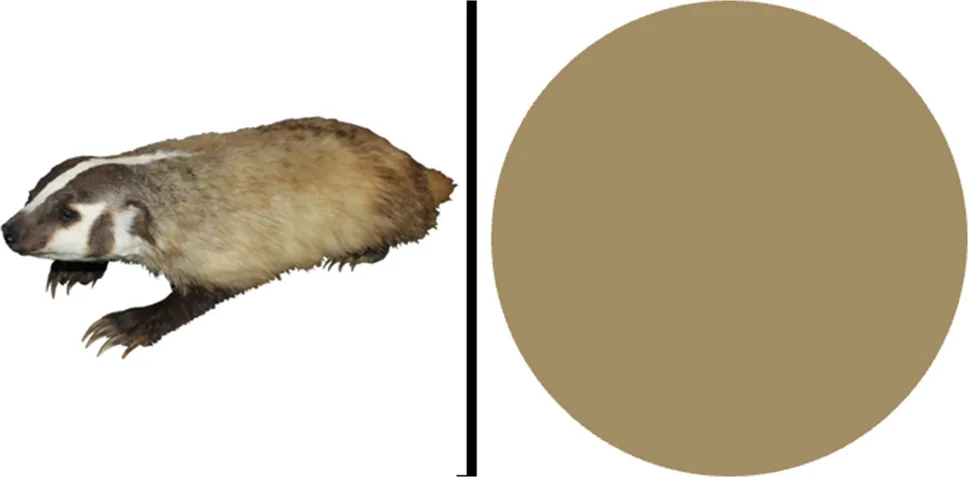
Target objects for Experiment 3. The badger on the left was used for participants assigned to search for real-world objects and the colored circle on the right was used for participants assigned to search for colored circles

While the design was still a 2 (stimulus type: real-world object, colored circle; between-subject) × 2 (target presence: present, absent; within-subject) × 3 (CDD: 15°, 30°, 45°; within-subject) mixed design, a single object was used for each stimulus type. For real-world objects, we used the MATCH toolbox to find a target object that could have many potential color-matching critical distractors such that no critical distractors repeated across the experiment. The chosen object was a badger shown in Fig. 12. For colored circles, the primary color of the badger was used as the single target color throughout the entire experiment (see Fig. 12). The primary color (L*, u*, v*) of both objects is (65, 17, 24).

#### Procedure

The task was identical to Experiment 2 except the 200-ms target presentation was removed. Participants were instructed and shown the target prior to the experimental task. Participants were told that they would be searching for the same target throughout the experiment.

### Results

Data were prepared as outlined in Experiments 1 and 2. One trial was removed due to eye movements suggestive of an anticipatory saccade and 30 trials for loss of eye movements.

#### Behavioral analysis

##### Accuracy

A 2 (stimulus type) × 2 (target presence) × 3 (CDD) mixed ANOVA for accuracy was conducted (see Table 7). Like Experiment 2, there was a significant main effect of stimulus type such that participants were more accurate when searching for a real-world object compared to a colored circle (*F*(1, 44) = 18.39, *p* < .001, η_p_^2^ = .29). When searching for a single consistent target, searching for a colored circle was more difficult than a real-world object. Surprisingly, we found a main effect of target presence such that participants were significantly more accurate on target-absent trials compared to target-present trials (*F*(1, 44) = 4.45, *p* = .04, η_p_^2^ = .09). There was also a significant main effect of color distance (*F*(1.08, 47.42) = 27.47, *p* < .001, η_p_^2^ = .38). We expected accuracy to be lower when CDD was smaller, as participants may have difficulty identifying the target when the critical distractor was so similar in color to the target. Post hoc tests using a Bonferroni correction with a corrected alpha of.017 were conducted and revealed that when CDD was small, the task was more difficult. Specifically, accuracy was significantly lower when the CDD was 15° compared to when it was 30° and 45° (all *p*s < .001). There were no significant differences in accuracy between 30° and 45° (*p* = .16).

**Table 7:** Means and standard deviations (*SD*s) of accuracies for Experiment 3

		Target presence			
		Target present		Target absent	
Stimulus type	Critical distractor distance	*M*	*Sd*	*M*	*SD*
Real-world object	15°	99.83%	0.55%	99.56%	0.85%
	30°	99.83%	0.58%	99.83%	0.55%
	45°	99.74%	0.69%	99.75%	0.88%
Colored circle	15°	85.09%	20.72%	85.69%	10.63%
	30°	88.76%	16.57%	99.66%	1.26%
	45°	87.68%	17.98%	99.75%	0.88%

We found several significant interactions: stimulus type and target presence (*F*(1, 44) = 4.64, *p* = .04, η_p_^2^ = .10), stimulus type and CDD (*F*(1.08, 47.42) = 26.30, *p* < .001, η_p_^2^ = .37), target presence and CDD (*F*(1.20, 52.92) = 31.32, *p* < .001, η_p_^2^ = .42), and a three-way interaction (*F*(1.20, 52.92) = 28.28, *p* < .001, η_p_^2^ = .39). Since we were interested in the differences between the stimulus types, to characterize the three-way interaction, we conducted 2 (stimulus type) × 3 (CDD) ANOVAs for target-present and target-absent trials separately.

For stimulus type and target presence, there were no significant differences in accuracy for real-world objects (*p* = 0.97, η_p_^2^ = .00002) but there was a significant difference for the colored circle condition (*F*(1, 44) = 9.09, *p* = .004, η_p_^2^ = .17) such that participants were more accurate on target-absent trials than target-present trials. The data provide further evidence that the participants likely had a difficult time discriminating between the target and the critical distractor.

Furthermore, the analysis for stimulus type and CDD, revealed that there was a significant effect for the colored circle condition (*F*(2, 43) = 28.22, *p* < .001, η_p_^2^ = .57) and we found evidence that participants indeed performed worse when the CDD was 15° compared to when it was 30° and 45° (all *p*s < .001). Participants likely had a difficult time discriminating between the critical distractor at CDD 15° and the target. We found no significant differences between CDD 30° and 45° (*p* = .27) and there were no significant differences in accuracy for real-world objects (*p* = .06, η_p_^2^ = .002).

For target presence and CDD, there were no significant differences in accuracy on target-present trials (*p* = .12, η_p_^2^ = .09) but there was a significant difference in accuracies on target-absent trials (*F*(2, 43) = 21.48, *p* < .001, η_p_^2^ = .50). When the CDD was 15°, participants were less accurate than when it was 30° and 45° (all *p*s < .001). Participants may have mistakenly identified the critical distractor at CDD 15° as the target. There were no differences in accuracy between CDDs of 30° and 45° on target-absent trials (*p* = 1.00).

For the three-way interaction, separate 2 (stimulus type) × 3 (CDD) ANOVAs for target-present and target-absent trials were conducted. This revealed a significant interaction on target-present trials (*F*(1.20, 52.95) = 4.01, *p* = .04, η_p_^2^ = .08) and target-absent trials (*F*(1.03, 45.19) = 39.68, *p* < .001, η_p_^2^ =.47). On target-present trials, participants were significantly more accurate when searching for a real-world object compared to a colored circle when the CDD was 15° (*F*(1, 44) = 11.63, *p* = .001, η_p_^2^ = .21), 30° (*F*(1, 44) = 10.25, *p* = .003, η_p_^2^ = .19), and 45° (*F*(1, 44) = 10.34, *p* = .002, η_p_^2^ = .19). On target-absent trials, we found that participants were also significantly more accurate when searching for a real-world object compared to a colored circle only when the CDD was 15° (*F*(1, 44) = 38.93, *p* < .001, η_p_^2^ = .47). There were no significant differences between stimulus types at CDDs 30° (*p* = .55, η_p_^2^ = .01) or 45° (*p* = 1.00, η_p_^2^ = 0.00). The three-way interaction demonstrated that across CDD, participants were consistently more accurate when searching for a real-world object compared to a colored circle on target-present trials. For target-absent trials, participants were only more accurate when searching for a real-world object when the CDD was 15°. Overall, the accuracy data indicates that searching for a real-world object was generally easier than searching for a colored circle.

##### Response times

**Fig. 13:**
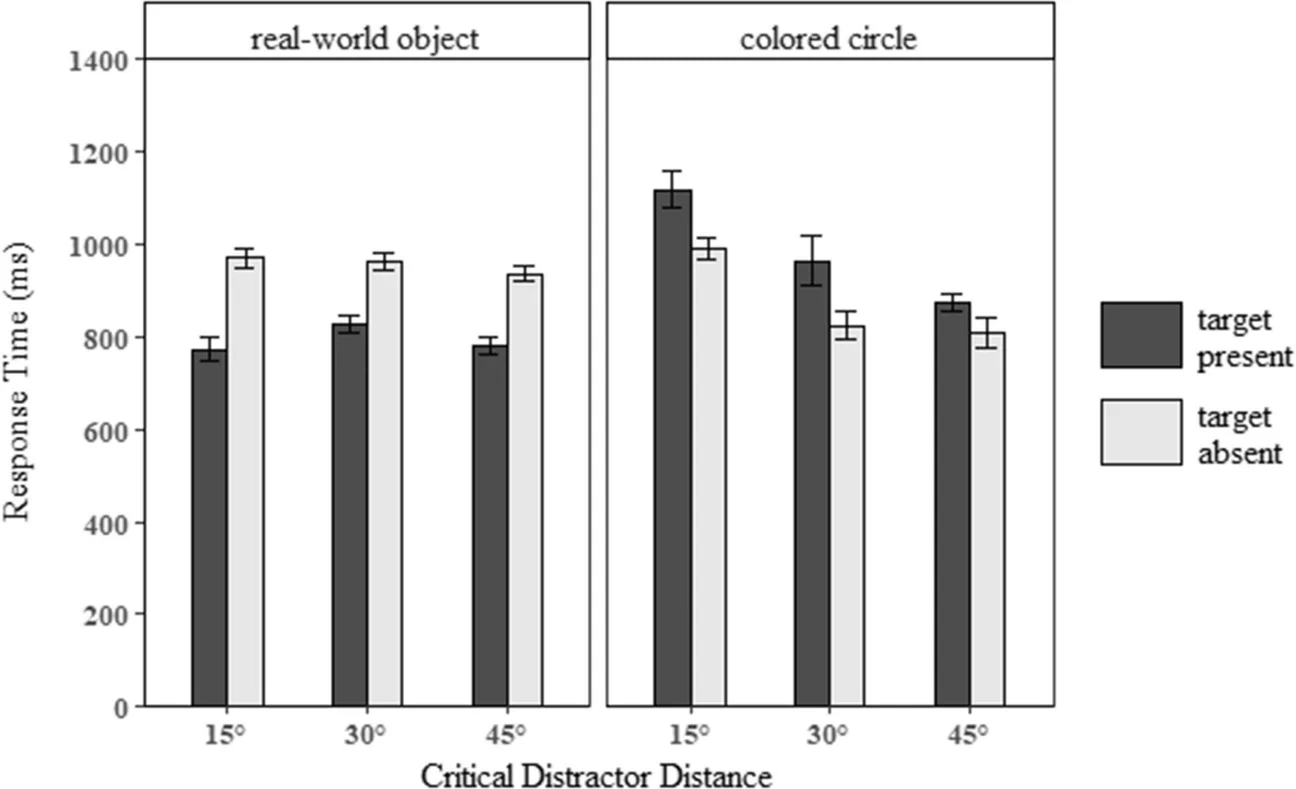
Response times for critical distractor distance by target presence for each stimulus type for Experiment 3. Error bars represent two within-subjects standard errors above and below the mean using the Cousineau-Morey method (Morey, 2008)

A 2 × 2 × 3 mixed ANOVA was conducted, and the mean RTs are shown in Fig. 13. We found no significant main effect of stimulus type (*p* = .54, η_p_^2^ = .008) or target presence (*p* = .37, η_p_^2^ = .02). We found a significant main effect of CDD (*F*(2, 88) = 23.93, *p* < .001, η_p_^2^ = .35). To further explore the main effect, we conducted post hoc tests with a Bonferroni correction using a corrected alpha of.017. This revealed that participants were significantly slower when the CDD was 15° compared to when it was 30° and 45° (all *p*s ≤ .003). Participants were also ~45 ms slower when the CDD was 30° compared to when it was 45° (*p* = .007). The results matched our predictions: smaller CDDs led to worse performance. A coarse guiding template likely directed attention to the critical distractors close in color to the target, thereby increasing RTs.

There was a significant interaction between stimulus type and target presence (*F*(1, 44) = 23.45, *p* < .001, η_p_^2^ = .34) and stimulus type and CDD (*F*(2, 88) = 22.88, *p* < .001, η_p_^2^ = .34). There were no significant interactions between target presence and CDD (*p* = .15, η_p_^2^ = .04) nor was there a significant three-way interaction (*p* =.13, η_p_^2^ =.05).

Overall, the results of the analysis between stimulus type and target presence indicated that participants were faster on target-present trials compared to target-absent trials, but the magnitude differed across stimulus types. We expected RTs to be faster on target-present trials as a longer duration is typically needed to search through the display and reject the possibility of the target’s presence. We found that there was a significant difference in RTs for the real-world object condition (*F*(1, 44) = 15.48, *p* < .001, η_p_^2^ = .27) such that participants were ~111 ms faster on target-present trials compared to target-absent trials. There was also a significant difference in RTs for the colored circle condition (*F*(1, 44) = 7.78, *p* = .008, η_p_^2^ = .15) such that participants were ~161 ms faster on target-absent trials compared to target-present trials.

For the significant interaction between stimulus type and CDD, there was no significant differences in RTs for real-world objects (*p* = .28, η_p_^2^ = .06) but there was for colored circles (*F*(2, 43) = 44.99, *p* < .001, η_p_^2^ = .68). Specifically, participants were significantly slower when the CDD was 15° compared to when it was 30° and 45° (all *p*s < .001), providing further evidence that participants likely had difficulty distinguishing between the target and critical distractor at CDD 15°. There were no differences in RTs between CDDs of 30° and 45° (*p* = .08).

#### Oculomotor analysis

Like Experiment 2, uninterpretable effects were not reported. This applies to the main effect of stimulus type, the main effect of CDD, and the interaction between stimulus type and CDD.

#### Target present

**Fig. 14:**
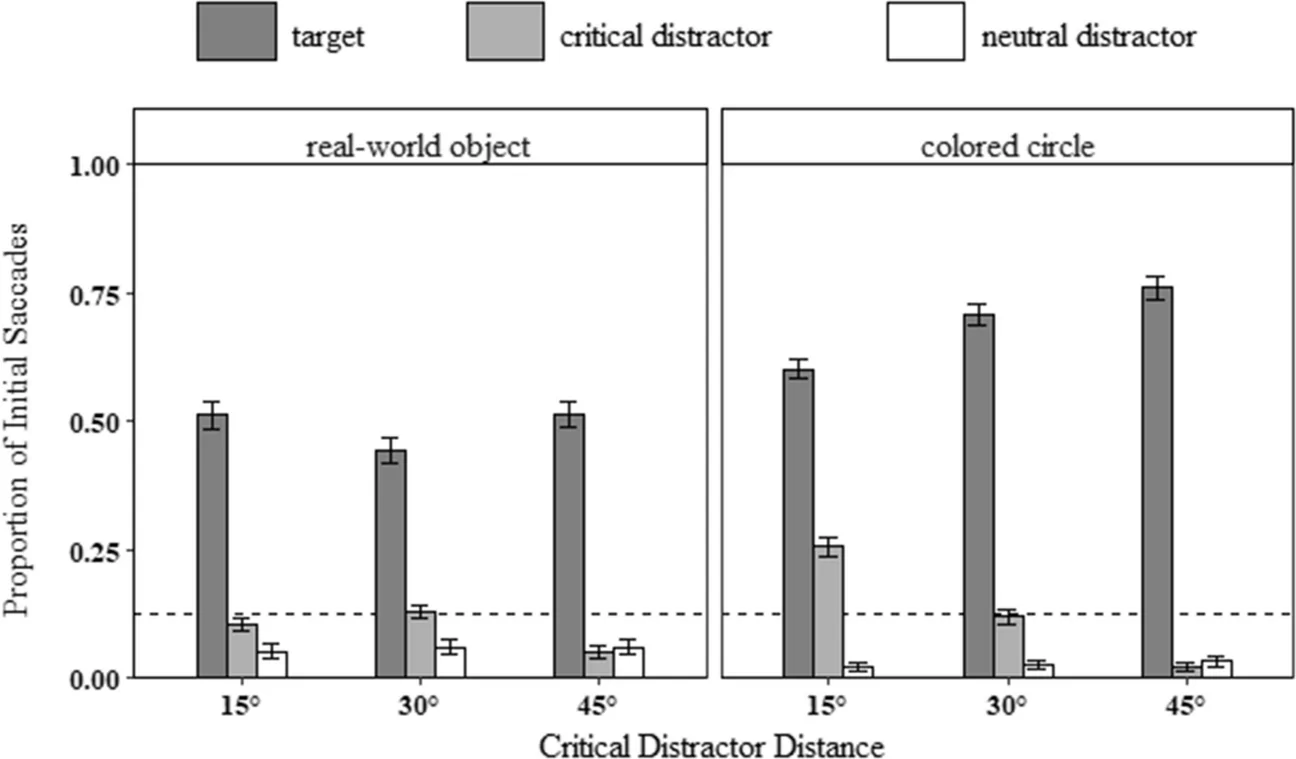
Proportion of initial saccades for object type by critical distractor distance on target present trials for each stimulus type for Experiment 3. The dotted line indicates the chance proportion if saccades were random (0.125). Error bars represent two within-subjects standard errors above and below the mean using the Cousineau-Morey method (Morey, 2008)

A 2 (stimulus type: real-world object, colored circle) × 3 (CDD: 15°, 30°, 45°) × 3 (object type: target, critical distractor, neutral distractor) mixed ANOVA for proportion of initial saccades to each object was conducted (see Fig. 14). We found a significant main effect of object type (*F*(1.11, 48.97) = 699.54, *p* < .001, η_p_^2^ = .94). To investigate the main effect of object type, post hoc tests with a Bonferroni correction using a corrected alpha of.017 were conducted and revealed that most initial saccades were directed to the target followed by the critical distractor and then the neutral distractor (all *p*s < .001). The results indicate that attention was highly efficient at locating the target though occasionally attention would be directed to the critical distractor.

We found several significant interactions: stimulus type and object type (*F*(1.11, 48.97) = 27.99, *p* < .001, η_p_^2^ = .39), CDD and object type (*F*(2.11, 93.05) = 43.96, *p* < .001, η_p_^2^ =.50), and a three-way interaction (*F*(2.11, 93.05) = 30.94, *p* < .001, η_p_^2^ = .41). To explore how guidance was affected across stimulus type and CDD, we conducted three separate 2 (stimulus type) × 3 (CDD) ANOVAs, one for each object type for the three-way interaction.

For stimulus type and object type it was revealed that there were significant differences in the proportion of initial saccades for the target (*F*(1, 44) = 30.81, *p* < .001, η_p_^2^ = .41), critical distractor (*F*(1, 44) = 13.57, *p* < .001, η_p_^2^ = .24), and neutral distractor (*F*(1, 44) = 23.05, *p* < .001, η_p_^2^ = .34). Specifically, for both the target and critical distractor, there was a significantly larger proportion of initial saccades to these objects when the stimulus type was colored circles compared to real-world objects. Meanwhile, the proportion of initial saccades to the neutral distractor were larger when searching for real-world objects compared to colored circles. Together, these results indicate that attention was guided to target-relevant objects when searching for a colored circle, but guidance was noisier when searching for a real-world object as initial saccades were directed to task-irrelevant objects.

For CDD and object type, we found that there was a significant difference in the proportion of initial saccades for the target (*F*(2, 43) = 16.92, *p* < .001, η_p_^2^ = .44), critical distractor (*F*(2, 43) = 142.75, *p* < .001, η_p_^2^ = .87), and neutral distractor (*F*(2, 43) = 10.89, *p* < .001, η_p_^2^ = .34). If the guiding template was coarse, we expected to see fewer saccades directed to critical distractors and, therefore, more saccades directed to the target at larger CDDs. The pattern of results supports our prediction and can be summarized as follows: when CDD increased, the proportion of initial saccades directed to the target increased but decreased for the proportion of initial saccades directed to the critical distractor and neutral distractor. Specifically, for initial saccades directed to the target, the proportion of initial saccades was significantly larger when the CDD was 45° compared to when it was 15° and 30° (all *p*s < .001). For initial saccades directed to the critical distractor, the proportion of initial saccades was significantly largest when the CDD was 15° followed by 30° and 45° then (all *p*s < .001). For initial saccades directed to the neutral distractor, the proportion of initial saccades was significantly smaller when the CDD was 15° compared to when it was 30° and 45° (all *p*s ≤ .02). All other comparisons between CDDs for the target and neutral distractors did not differ (all *p*s ≥ .10).

For the three-way interaction, we conducted a 2 (stimulus type) × 3 (CDD) ANOVA for each object type. There was a significant interaction for when the initial saccade was directed to the target (*F*(1.77, 77.80) = 20.80, *p* < .001, η_p_^2^ = .32) and critical distractor (*F*(1.59, 69.76) = 41.61, *p* < .001, η_p_^2^ = .49). There was no significant interaction for the neutral distractor (*p* = .24, η_p_^2^ = .03). There was a significantly smaller proportion of initial saccades to the target when the stimulus type was a real-world object compared to a colored circle at CDDs 15° (*F*(1, 44) = 5.36, *p* = .03, η_p_^2^ = .11), 30° (*F*(1, 44) = 45.44, *p* < .001, η_p_^2^ = .51), and 45° (*F*(1, 44) = 34.38, *p* < .001, η_p_^2^ = .44). When the initial saccades were directed to the critical distractor, the proportion was smaller when searching for real-world objects compared to colored circles at CDD 15° (*F*(1, 44) = 58.42, *p* < .001, η_p_^2^ = .57). Conversely, at CDDs 45° (*F*(1, 44) = 13.05, *p* < .001, η_p_^2^ = .23) the proportion was of initial saccades that were directed to the critical distractor was larger when searching for a real-world object compared to a colored circle. There were no significant differences in the proportion of initial saccades to the critical distractor at CDD 30° between stimulus types (*p* = .51, η_p_^2^ = .01). The results of the three-way interaction indicate that attention was directed more to target-relevant areas when searching for a colored-circle compared to a real-world object. This is evidenced by the higher frequency of saccades to the target and the critical distractor at CDD 15° in the colored circle condition. Though we found a slight but significantly larger proportion of saccades to the critical distractor at CDD 45° when searching for real-world objects compared to colored circles, the proportion must be compared to the random chance of saccading to the object to provide supporting evidence that the guiding template for a real-world object is coarser than a colored circle.

One-tailed *t*-tests comparing each proportion of initial saccades to chance (0.125) (see Table 8) revealed that the proportion of initial saccades directed to the critical distractor at CDD 45° was not significantly greater than chance for the real-world object condition (*p* = 1.00). However, the proportions of initial saccades directed to the target at all CDDs (15°, 30°, and 45°) in the real-world objects condition, the target at all CDDs (15°, 30°, and 45°) in the colored circles condition, and the critical distractor at CDD 15° in the colored circle condition were all above chance (all *p*s < .001). All other proportions were not significant (all *p*s ≥ .31). The results provide supporting evidence that attention was directed more towards target-relevant areas, specifically the target and critical distractor at CDD 15°, when searching for the colored circles compared to real-world objects.

**Table 8:** One-tailed *t*-test results comparing proportion of initial saccades on target present trials to chance in Experiment 3

Stimulus type	Object type	Critical distractor distance	Test	Statistic	df	*p*-value	Effect size
Real-world objects	Target	15°	Student	11.51	22	<.001	2.40
		30°	Student	9.95	22	<.001	2.08
		45°	Wilcoxon	275		<.001	.99
	Critical distractor	15°	Student	−2.25	22	.98	−0.47
		30°	Student	0.50	22	.31	0.10
		45°	Wilcoxon	2		>.99	-.99
	Neutral distractor	15°	Student	−13.01	22	>.99	−2.71
		30°	Student	−11.17	22	>.99	−2.33
		45°	Student	−10.92	22	>.99	−2.28
Colored circles	Target	15°	Student	25.13	22	<.001	5.24
		30°	Student	25.47	22	<.001	5.31
		45°	Student	24.61	22	<.001	5.13
	Critical distractor	15°	Student	7.40	22	<.001	1.54
		30°	Student	−0.45	22	.67	−0.09
		45°	Wilcoxon	0		>.99	<-.99
	Neutral distractor	15°	Student	−38.57	22	>.99	−8.04
		30°	Student	−27.64	22	>.99	−5.76
		45°	Student	−20.60	22	>.99	−4.29

The effect size reported for a Student’s *t*-test was Cohen’s *d.* The effect size reported for a Wilcoxon test was rank-biserial correlation (*r*)

#### Target absent

**Fig. 15:**
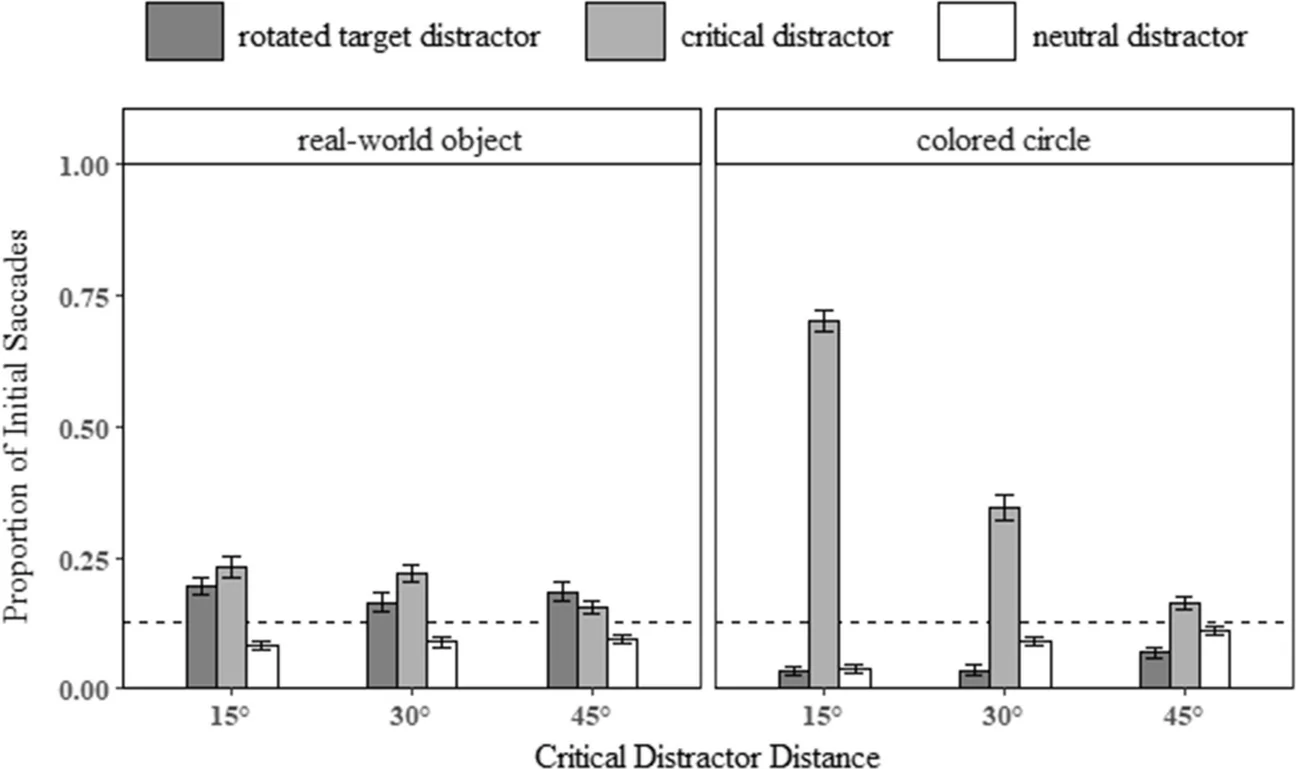
Proportion of initial saccades for object type by critical distractor distance on target-absent trials for each stimulus type for Experiment 3. The dotted line indicates the chance proportion if saccades were random (0.125). Error bars represent two within-subjects standard errors above and below the mean using the Cousineau-Morey method (Morey, 2008)

The mean proportions of initial saccades for target-absent trials are presented in Fig. 15. We conducted a 2 (stimulus type: real-world object, colored circle) × 3 (CDD: 15°, 30°, 45°) × 3 (object type: rotated target distractor, critical distractor, neutral distractor) mixed ANOVA for proportion of initial saccades to each object. We found a significant main effect of object type (*F*(1.59, 69.86) = 174.27, *p* < .001, η_p_^2^ = .80). Post hoc tests were conducted with a Bonferroni correction using a corrected alpha of.017. We found that a significantly larger proportion of initial saccades to the critical distractor compared to the rotated target distractor and neutral distractor (all *p*s < .001). When compared to the corrected alpha, there was no significant difference in the proportion of initial saccades between the rotated target distractor and neutral distractor (*p* = .03). The results indicate that when searching for a single object, color was the predominant feature used to guide attention, but other feature dimensions cannot be used to guide attention in the colored circle condition. Therefore, further analyses of the interactions are required.

There were several significant interactions: stimulus type and object type (*F*(1.59, 69.86) = 88.66, *p* < .001, η_p_^2^ = .67), CDD and object type (*F*(2.78, 122.27) = 138.29, *p* < .001, η_p_^2^ = .76), and a three-way interaction (*F*(2.78, 122.27) = 86.77, *p* < .001, η_p_^2^ = .66). To examine the effects of guidance across stimulus type and CDD, separate 2 (stimulus type) × 3 (CDD) ANOVAs for each object type were conducted to further characterize the three-way interaction.

For stimulus type and object type, there was a significant difference in the proportion of initial saccades for the critical distractor (*F*(1, 44) = 67.61 *p* < .001, η_p_^2^ = .61) and rotated target distractor (*F*(1, 44) = 62.12, *p* < .001, η_p_^2^ = .59). There was no significant difference in the proportion of initial saccades that were directed to neutral distractors (*p* = .22, η_p_^2^ = .04). Specifically, there was a smaller proportion of initial saccades to the critical distractor when participants were searching for a real-world object compared to a colored circle but there was a larger proportion of initial saccades to the rotated target distractor when searching for a real-world object compared to a colored circle. The results provide evidence that other feature dimensions were used to guide attention in the real-world object condition.

For CDD and object type, we found a significant difference in the proportion of initial saccades that were directed to the critical distractor (*F*(2, 43) = 181.88, *p* < .001, η_p_^2^ = .89), rotated target distractor (*F*(2, 43) = 4.66, *p* = .01, η_p_^2^ = .18), and neutral distractor (*F*(2, 43) = 128.03, *p* < .001, η_p_^2^ = .86). The proportion of initial saccades directed to the critical distractor was largest at CDD 15° followed by 30° and then 45° (all *p*s < .001). Conversely, the proportion of initial saccades directed to the neutral distractor was largest at CDD 45° followed by 30° and then 15° (all *p*s < .001). For initial saccades directed to the rotated target distractor, we found a significantly larger proportion of initial saccades to the rotated target distractor when the CDD was 45° compared to when it was 30° (*p* = .01). All other comparisons for the proportion of initial saccades to the rotated target distractor were not significant (all *p*s ≥ .43). Together, the interaction between CDD and object type indicates that the guiding template may be coarse, but the range of color used to guide attention may be smaller than 45° from the target’s veridical color. If no object shared this broad range of colors, it appeared that other, more efficient target features may have been used to guide attention as evidenced by more initial saccades directed to the rotated target distractor at large CDDs. However, this analysis collapses across stimulus conditions. In the colored circle condition, other feature dimensions cannot be used to guide attention. Therefore, analysis of the three-way interaction is required.

For the three-way interaction we conducted separate 2 (stimulus type) × 3 (CDD) ANOVA for each object type. We found a significant interaction for initial saccades directed to the critical distractor (*F*(2, 88) = 135.26, *p* < .001, η_p_^2^ = .75) and neutral distractor (*F*(2, 88) = 71.18, *p* < .001, η_p_^2^ = .62), but not for the rotated target distractor (*p* = .09, η_p_^2^ = .06). We found a significantly smaller proportion of initial saccades that were directed to the critical distractor when the stimulus type was a real-world object compared to a colored circle at CDDs 15° (*F*(1, 44) = 222.54, *p* < .001, η_p_^2^ = .84) and 30° (*F*(1, 44) = 12.72, *p* < .001, η_p_^2^ = .22). There was no significant difference in the proportion of initial saccades to the critical distractor across stimulus types when the CDD was 45° (*p* = .81, η_p_^2^ = .001). For initial saccades that were directed to the neutral distractor, the proportion was larger when searching for a real-world object compared to a colored circle at CDD 15° (*F*(1, 44) = 39.60, *p* < .001, η_p_^2^ = .47) but the proportion was smaller when searching for real-world objects compared to colored circles at CDD 45° (*F*(1, 44) = 5.25, *p* = .03, η_p_^2^ = .11). There was no significant difference in the proportion of initial saccades to the neutral distractor across stimulus types when the CDD was 30° (*p* = .80, η_p_^2^ = .002). In the absence of the target, we found increased saccade priority for the critical distractor in the colored circle condition compared to real-world object condition. The previously analyzed stimulus condition and object type interaction and CDD and object type interaction implied that when searching for real-world objects, attention was directed to the rotated target distractor at large CDDs. Therefore, we provisionally speculated that color and other feature dimensions may have been used equivalently to guide search for real-world objects. Surprisingly, the three-way interaction provides evidence against this. Though the proportion of initial saccades to the rotated target distractor was relatively larger at each CDD in the real-world objects condition, there was no significant difference between stimulus types. Furthermore, we did not find evidence that the guiding template was coarser for real-world objects than for colored circles. The proportion of initial saccades to the critical distractor when the CDD was 45° was similar across stimulus types. To provide further evidence that there was no guidance to these distractors, we conducted two additional analyses described below. First, we examined whether the proportion of initial saccades to the critical distractor when the CDD was 45° (in either stimulus condition) was larger than the chance of saccading to each object. Second, for each stimulus condition, we compared the proportion of initial saccades to the critical distractor when the CDD was 45° to neutral distractors.

The results of the one-tailed *t*-tests comparing each proportion of initial saccades to chance (0.125) are presented in Table 9. We found that the proportion of initial saccades directed to the critical distractor at all CDDs (15°, 30°, and 45°) in the real-world objects condition, the critical distractor at all CDDs (15°, 30°, and 45°) in the colored circles condition, and the rotated target distractor at all CDDs (15°, 30°, and 45°) in the real-world objects condition were greater than chance (all *p*s ≤ .04). All other proportions were not significant (all *p*s > .99). The results provide additional evidence that feature dimensions, other than color, were used to guide search for a real-world object. Additionally, the results suggest that there was guidance to the critical distractors at CDD 45° for both stimulus conditions. While this may indicate that guidance was coarser than expected, we compared the proportion of initial saccades to the critical distractor at CDD 45° to neutral distractors below.

**Table 9:** One-tailed *t*-test results comparing proportion of initial saccades on target-absent trials to chance in Experiment 3

Stimulus type	Object type	Critical distractor distance	Test	Statistic	*df*	*p*-value	Effect size
Real-world objects	Rotated target distractor	15°	Student	4.06	22	<.001	0.85
		30°	Student	1.88	22	.04	0.39
		45°	Wilcoxon	232		<.001	.68
	Critical distractor	15°	Student	4.89	22	<.001	1.02
		30°	Student	4.92	22	<.001	1.03
		45°	Student	2.29	22	.02	0.48
	Neutral distractor	15°	Student	−7.54	22	>.99	−1.57
		30°	Student	−6.21	22	>.99	−1.29
		45°	Wilcoxon	0		>.99	<-.99
Colored circles	Rotated target distractor	15°	Student	−16.90	22	>.99	−3.52
		30°	Wilcoxon	0		>.99	<-.99
		45°	Student	−8.25	22	>.99	−1.72
	Critical distractor	15°	Student	25.70	22	<.001	5.36
		30°	Student	7.67	22	<.001	1.60
		45°	Student	2.34	22	.001	0.49
	Neutral distractor	15°	Student	−23.80	22	>.99	−4.96
		30°	Student	−7.59	22	>.99	−1.58
		45°	Student	−3.38	22	>.99	−0.70

The effect size reported for a Student’s *t*-test was Cohen’s *d.* The effect size reported for a Wilcoxon test was rank-biserial correlation (*r*)

#### 45° critical distractor versus neutral distractor

To provide further evidence that a coarser template was employed, we examined the interaction between stimulus type (real-world object, colored circle) and object type (critical distractor, neutral distractor). We focused on only target-absent trials since most initial saccades were directed to the target on target-present trials. The interaction was not significant (*p* = .58, η_p_^2^ = .01). Combined with the analysis evaluating whether the proportion of initial saccades to the critical distractor at CDD 45° were above chance, we speculate that the guiding template may occasionally be guided to colors far from the target but overall attention prioritized the critical distractors at CDD 45° and neutral distractors similarly. Critically, these analyses provide evidence that the results from Experiments 1 and 2 could be due to a poor representation of the target object in long-term memory leading to a coarser representation for a guiding template. However, we did not find evidence that coarser templates were used when searching for a real-world object in comparison to a simpler stimulus. Rather, we found evidence that coarser templates are employed across stimulus types. We previously expected that a coarser template would be used when searching for real-world objects to adapt to changing search contexts (e.g., kitchen versus living room) and lighting conditions. We expected a larger range of colors to guide attention; however, real-world objects can be defined by other feature dimensions. It is possible that rather than relying on a larger range of colors to orient attentional mechanisms, guidance may instead use a broader range of feature dimensions.

## General discussion

The goal of our studies was to demonstrate whether a coarse guiding template is utilized when searching for real-word objects. In Experiments 1 and 2, we found evidence that a coarse guiding template was used as attention was oftentimes misguided to distractors that were close in color distance to the target’s primary color. However, unlike previous research (Yu et al., 2022), attention was directed toward distractors that were well beyond the 10–29° rotation from the target’s veridical color. We initially hypothesized that coarser guiding templates may be employed due to their benefits in the real-world. Oftentimes, real-world objects are not uniformly defined by an isolated color. Additionally, color perception can vary due to differences in lighting or the contextual surroundings. However, the results from Experiment 2 indicated that a template coarser than the 10–29° rotation from the target’s veridical color was employed for both colored circles and real-world objects. Therefore, a more likely factor was the inability to form a precise target template in long-term memory. Across our Experiments 1 and 2, the target object switched trial-to-trial, preventing an accurate representation from forming (e.g., Bravo & Farid, 2012; Goldstein & Beck, 2018). In Experiment 3, we used a constant target across both stimulus types (real-world objects, colored circles) and found more similar results to previous findings (Yu et al., 2022). The results from Experiment 3 demonstrated that coarse guiding templates are employed for both complex, real-world objects and simpler, colored circles.

Although color is frequently prioritized as the guiding feature (Alexander et al., 2019; Motter & Belky, 1998; Rutishauser & Koch, 2007), our data suggest that other features, such as shape, could be used to guide attention when observers are tasked with searching for real-world objects. We found evidence that attention was often guided to the object that matched in shape but not color, indicating that observers made use of, at least to some degree, non-color features to guide attention. The use of other features can be further supported by the attenuated inversely proportional relationship between CDD and the proportion of initial saccades directed to the critical distractor for real-world objects. Indeed, the proportion of initial saccades directed to the critical distractor was larger at almost every CDD for colored circles compared to real-world objects. If color was the only feature used to guide attention, we would have expected similar proportions across the two stimulus conditions. Consistent with the good-enough guidance principle, while the feature used may not be veridical to the target features, the most efficient feature is often used to guide attention (Yu et al., 2023). Some observers may also have discovered that by guiding attention to the shape-matching object, they would only have to verify whether it was the correct color. If the colors matched the target, it was a target-present trial; otherwise, it was a target-absent trial. It is important to note that we only presented distractors that were potentially in the color space range of the broad guiding template and there were no equivalent distractors for shape information. In other words, we did not control the shape range. The observer’s use of an alternative search strategy may have hindered our ability to find evidence that coarser guiding templates are used when searching for a real-world object in comparison to colored circles. Further research should attempt to isolate the effects of shape. Additionally, future research should investigate whether other types of features, like shape, are also represented non-veridically in the guiding template. We would expect that since color was represented broadly, so too might other feature dimensions.

While Yu and colleagues (2022) suggested that the guiding template may be 10–29° coarser than the target’s veridical color, their search conditions slightly differed from ours. Both studies presented one single critical distractor, however our study included target-absent trials which were used to critically inform the coarseness of the guiding template. On target-absent trials, we presented another target-similar distractor in the real-world objects condition: the rotated target distractor. We did not originally consider the shape-matching object as a “critical distractor.” As such, we may have unintentionally created a somewhat different search context from the one presented by Yu and colleagues (2022) such that there would be two “critical distractors” on half of the trials. As a result, we cannot conclude whether the range of feature values proposed by Yu and colleagues (2022) extends directly to other search contexts. Regardless, the results of our studies indicate that a coarser template is used for both simple circles and complex real-world objects, even if we are unable to explicitly define the range of feature values encapsulated within that template. To that point, the range of feature values within the guiding template may be highly dependent on the search context.

Another difference between previous work and our study is that information used during guidance was compared to the information used during the identification of the target (Yu et al., 2022). Our study makes no such comparison, as our goal was to provide further evidence that a broad guiding template was employed during search. Without this comparison, it is possible that our results may be better explained by distractor similarity (Duncan & Humphreys, 1989). However, the classical findings of distractor similarity may be due, in part, to the coarseness of guiding templates. If the guiding template held a more precise range of feature values, we would have expected little guidance to the critical distractor on target-present trials. In our study, we observed that a significant proportion of initial saccades were directed to the critical distractor even when the target was present. In conclusion, our results provide some evidence against a target-distractor similarity account, but future research should investigate the contents of the guiding template compared to the target template.

The present study highlights that while a coarser template may be inherent to guiding attention for various practical benefits (see Yu et al., 2023, for a review), other factors may affect its coarseness. Many studies examining target template specificity typically focus on the effects of semantic versus visual cues or on the effects of image manipulations on the visual cue (Bravo & Farid, 2009; Malcom & Henderson, 2009; Vickery et al., 2005; Wolfe & Horowitz, 2004). In these studies, the visual cue that matches the target object typically guides attention most efficiently. We found evidence that, despite presenting an exact visual cue, the target information did not appear to be perfectly represented. In other words, the information was best represented when a single consistent target was presented. Our results support the recent findings that the stronger memory representation guided attention best (Williams et al., 2022). It should be noted that the delineation we used is one possible mechanism for how target information is represented (i.e., target template held in long-term memory, guiding template held in working memory) (Wolfe, 2021). Templates are a construct and how information is represented in the brain may be more nuanced than the strict characterizations described here. Regardless, guidance may be strongly affected by how the information is represented in memory.

## Conclusion

Across three experiments, we found evidence that a coarser representation of the target is used to guide attention regardless of the identity of the target. In other words, whether the target was a circle or a potato, the guiding template does not use a veridical representation to guide attention. We also demonstrated that the magnitude of coarseness within a guiding template appears to be affected by how well the information is represented in memory.

## Data Availability

The three experiments described in the article were preregistered and the data are available in the Open Science Framework repository (https://doi.org/10.17605/OSF.IO/PBG4U).

## Footnotes

1 The one-tailed *t*-tests comparing the proportion of initial saccade to each object was not originally part of the preregistration. Accordingly, the analysis should be considered exploratory.

## References

[CR1] Alexander, R. G., Nahvi, R. J., & Zelinsky, G. J. (2019). Specifying the precision of guiding features for visual search. *Journal of Experimental Psychology: Human Perception and Performance,**45*(9), 1248–1264. 10.1037/xhp0000668 PMC6706321 31219282

[CR2] Brady, T. F., Konkle, T., Alvarez, G. A., & Oliva, A. (2008). Visual long-term memory has a massive storage capacity for object details. *Proceedings of the National Academy of Sciences,**105*(38), 14325–14329. 10.1073/pnas.0803390105 PMC2533687 18787113

[CR3] Brady, T. F., Konkle, T., Alvarez, G. A., & Oliva, A. (2013). Real-world objects are not represented as bound units: independent forgetting of different object details from visual memory. *Journal of Experimental Psychology: General,**142*(3), 791–808. 10.1037/a0029649 22905874

[CR4] Bravo, M. J., & Farid, H. (2009). The specificity of the search template. *Journal of Vision,**9*(1), 34–34. 10.1167/9.1.34 19271904

[CR5] Bravo, M. J., & Farid, H. (2012). Task demands determine the specificity of the search template. *Attention, Perception, & Psychophysics,**74*(1), 124–131. 10.3758/s13414-011-0224-5 22006527

[CR6] Brodeur, M. B., Guérard, K., & Bouras, M. (2014). Bank of standardized stimuli (BOSS) phase II: 930 new normative photos. *PloS One,**9*(9), Article e106953. 10.1371/journal.pone.0106953 PMC4161371 25211489

[CR7] Carlisle, N. B., Arita, J. T., Pardo, D., & Woodman, G. F. (2011). Attentional templates in visual working memory. *Journal of Neuroscience,**31*(25), 9315–9322. 10.1523/JNEUROSCI.1097-11.2011 PMC3147306 21697381

[CR8] Duncan, J., & Humphreys, G. W. (1989). Visual search and stimulus similarity. *Psychological Review,**96*(3), 433–458. 10.1037/0033-295X.96.3.433 2756067

[CR9] Goetz, J. N., & Neider, M. B. (2025a). MATCH: A toolbox to assess the primary color of real-world objects and generate color-matching stimuli. *Behavior Research Methods,**57*(12), 1–22. 10.3758/s13428-025-02856-3 41162664

[CR10] Goetz, J. N., & Neider, M. B. (2025b). Proportion of colour proportionally affects guidance in search for conjunction and real-world objects. *Visual Cognition,**33*(1), 42–66. 10.1080/13506285.2025.2493945

[CR11] Goldstein, R. R., & Beck, M. R. (2018). Visual search with varying versus consistent attentional templates: Effects on target template establishment, comparison, and guidance. *Journal of Experimental Psychology: Human Perception and Performance,**44*(7), 1086–1102. 10.1037/xhp0000520 29620379

[CR12] Henderson, J. M., Pollatsek, A., & Rayner, K. (1989). Covert visual attention and extrafoveal information use during object identification. *Perception & Psychophysics,**45*(3), 196–208. 10.3758/bf03210697 2710617

[CR13] Konkle, T., Brady, T. F., Alvarez, G. A., & Oliva, A. (2010). Conceptual distinctiveness supports detailed visual long-term memory for real-world objects. *Journal of Experimental Psychology: General,**139*(3), 558–578. 10.1037/a0019165 PMC3398125 20677899

[CR14] Malcom, G. L., & Henderson, J. M. (2009). The effects of target template specifi city on visual search in real-world scenes: Evidence from eye movements. *Journal of Vision*, *9*(11). 10.1167/9.11.8 20053071

[CR15] Morey, R. D. (2008). Confidence intervals from normalized data: A correction to Cousineau (2005). *Tutorials in Quantitative Methods for Psychology,**4*(2), 61–64. 10.20982/tqmp.04.2.p061

[CR16] Motter, B. C., & Belky, E. J. (1998). The guidance of eye movements during active visual search. *Vision Research,**38*(12), 1805–1815. 10.1016/S0042-6989(97)00349-0 9797959

[CR17] Oliva, A., Torralba, A., Castelhano, M. S., & Henderson, J. M. (2003). Top-down control of visual attention in object detection. *Proceedings 2003 international conference on image processing (Cat. No. 03CH37429)* (vol. 1, p. I–253). IEEE.

[CR18] Peirce, J., Gray, J. R., Simpson, S., MacAskill, M., Höchenberger, R., Sogo, H., ..., & Lindeløv, J. K. (2019). PsychoPy2: Experiments in behavior made easy. *Behavior research methods,**51*(1), 195–203. 10.3758/s13428-018-01193-y PMC6420413 30734206

[CR19] Rutishauser, U., & Koch, C. (2007). Probabilistic modeling of eye movement data during conjunction search via feature-based attention. *Journal of Vision,**7*(6), 5. 10.1167/7.6.5 17685788

[CR20] Scialfa, C. T., & Joffe, K. M. (1998). Response times and eye movements in feature and conjunction search as a function of target eccentricity. *Perception & Psychophysics,**60*(6), 1067–1082. 10.3758/BF03211940 9718964

[CR21] Vickery, T. J., King, L.-W., & Jiang, Y. (2005). Setting up the target template in visual search. *Journal of Vision,**5*(1), 8. 10.1167/5.1.8 15831069

[CR22] Wickham, H. (2016). *Ggplot2: Elegant graphics for data analysis*. Springer-Verlag.

[CR23] Williams, D. E., Reingold, E. M., Moscovitch, M., & Behrmann, M. (1997). Patterns of eye movements during parallel and serial visual search tasks. *Canadian Journal of Experimental Psychology/Revue Canadienne de Psychologie Expérimentale,**51*(2), 151–164. 10.1037/1196-1961.51.2.151 9340075

[CR24] Williams, J. R., Brady, T. F., & Störmer, V. S. (2022). Guidance of attention by working memory is a matter of representational fidelity. *Journal of Experimental Psychology: Human Perception and Performance,**48*(3), 202–231. 10.1037/xhp0000985 35084932

[CR25] Wolfe, J. M. (2021). Guided search 6.0: An updated model of visual search. *Psychonomic Bulletin & Review,**28*(4), 1060–1092. 10.3758/s13423-020-01859-9 PMC8965574 33547630

[CR26] Wolfe, J. M., & Horowitz, T. S. (2004). What attributes guide the deployment of visual attention and how do they do it? *Nature Reviews Neuroscience,**5*(6), 495–501. 10.1038/nrn1411 15152199

[CR27] Wolfe, J. M., & Horowitz, T. S. (2017). Five factors that guide attention in visual search. *Nature Human Behaviour*. 10.1038/s41562-017-0058 PMC9879335 36711068

[CR28] Wolfe, J. M., Horowitz, T. S., Kenner, N., Hyle, M., & Vasan, N. (2004). How fast can you change your mind? The speed of top-down guidance in visual search. *Vision Research,**44*(12), 1411–1426. 10.1016/j.visres.2003.11.024 15066400

[CR29] Yu, X., Johal, S. K., & Geng, J. J. (2022). Visual search guidance uses coarser template information than target-match decisions. *Attention, Perception, & Psychophysics,**84*(5), 1432–1445. 10.3758/s13414-022-02478-3 PMC9232460 35474414

[CR30] Yu, X., Zhou, Z., Becker, S. I., Boettcher, S. E., & Geng, J. J. (2023). Good-enough attentional guidance. *Trends in Cognitive Sciences,**27*(4), 391–403. 10.1016/j.tics.2023.01.007 36841692

[CR31] Zelinsky, G. J. (2008). A theory of eye movements during target acquisition. *Psychological Review,**115*(4), 787–835. 10.1037/a0013118 PMC2577318 18954205

